# Skeletal muscle endothelial dysfunction through the activin A–PGC1α axis drives progression of cancer cachexia

**DOI:** 10.1038/s43018-025-00975-6

**Published:** 2025-05-26

**Authors:** Young-Mee Kim, Mark A. Sanborn, Shaluah Vijeth, Priyanka Gajwani, Xinge Wang, Dahee Jung, Tibor Valyi-Nagy, Sreeparna Chakraborty, Georgina Mancinelli, Peter T. Toth, Evan H. Phillips, Paul Grippo, Ameen A. Salahudeen, Jooman Park, Su Yeon Yeon, Vijayalakshmi Ananthanarayanan, Yuwei Jiang, Steve Seung-Young Lee, Klara Valyi-Nagy, Jalees Rehman

**Affiliations:** 1 Department of Biochemistry and Molecular Genetics, University of Illinois College of Medicine, Chicago, IL, USA.; 2 University of Illinois Cancer Center, Chicago, IL, USA.; 3 Department of Pharmaceutical Sciences, University of Illinois College of Pharmacy, Chicago, IL, USA.; 4 Department of Pathology, University of Illinois College of Medicine, Chicago, IL, USA.; 5 Division of Gastroenterology and Hepatology, Department of Medicine, University of Illinois College of Medicine, Chicago, IL, USA.; 6 Research Resources Center, University of Illinois Chicago, Chicago, IL, USA.; 7 Division of Hematology and Oncology, Department of Medicine, University of Illinois College of Medicine, Chicago, IL, USA.; 8 Department of Physiology and Biophysics, University of Illinois College of Medicine, Chicago, IL, USA.

## Abstract

Cachexia is the wasting of skeletal muscle in cancer and is a major complication that impacts a person’s quality of life. We hypothesized that cachexia is mediated by dysfunction of the vascular system, which is essential for maintaining perfusion and tempering inappropriate immune responses. Using transparent tissue topography, we discovered that loss of muscle vascular density precedes muscle wasting in multiple complementary tumor models, including pancreatic adenocarcinoma, colon carcinoma, lung adenocarcinoma and melanoma models. We also observed that persons suffering from cancer cachexia exhibit substantial loss of muscle vascular density. As tumors progress, increased circulating activin A remotely suppresses the expression of peroxisome proliferator-activated receptor-γ coactivator 1α (PGC1α) in the muscle endothelium, thus inducing vascular leakage. Restoring endothelial PGC1α activity preserved vascular density and muscle mass in tumor-bearing mice. Our study suggests that restoring muscle endothelial function could be a valuable therapeutic approach for cancer cachexia.

Cachexia refers to the skeletal muscle loss that is observed in multiple chronic diseases including cancer^1^. This wasting of skeletal muscle greatly impairs the activities of daily living and is a key factor in determining the quality of life^2^. Despite its prevalence in advanced chronic diseases, our understanding of the mechanistic underpinnings of cachexia remains inadequate, which may in part explain the dearth of treatments approved by the US Food and Drug Administration^2,3^ and only one approved therapy in Japan^4^. Recent research has focused on the impact of inflammatory cytokines as likely mediators of cachexia^3,5^. These findings raise intriguing mechanistic questions of how circulating inflammatory mediators enter the muscle to promote cachexia and how additional cell populations or mediators contribute to cachexia.

The inner walls of blood vessels are lined with endothelial cells (ECs), which are directly exposed to circulating immune cells, nutrients, cytokines and other small molecules in the blood^6,7^. ECs sense changes in circulating factors and thereby provide cues to regulate tissue homeostasis^8^. As skeletal muscle is a highly vascularized tissue^9^, the role of the muscle vasculature in mediating cachexia needs to be investigated. Aging in humans is associated with a loss of muscle vasculature, which correlates with a decrease in muscle mass and muscle strength^10,11^, mirroring key features of cancer cachexia^12^ and, thus, suggesting that similar mechanisms could be at play. Interestingly, cachexic muscles are often spatially removed from active disease sites, indicating that cachexic factors are released from disease sites and act remotely on the muscle through blood circulation. This highlights the importance of ECs, the first point of contact for circulating factors in the blood.

Here we hypothesized that endothelial dysfunction in skeletal muscles mediates cancer cachexia. Using four complementary in vivo tumor models, we investigated the temporal and mechanistic relationship between skeletal muscle endothelial dysfunction and muscle loss in cancer cachexia and whether circulating cachexic cues induce muscle vascular dysfunction. Furthermore, we examined the benefits of therapeutically restoring muscle endothelial function on cachexia progression in tumor-bearing mice.

## Results

### Reduced vascular density in cachexic muscles

**Extended Data Fig. 1 |:**
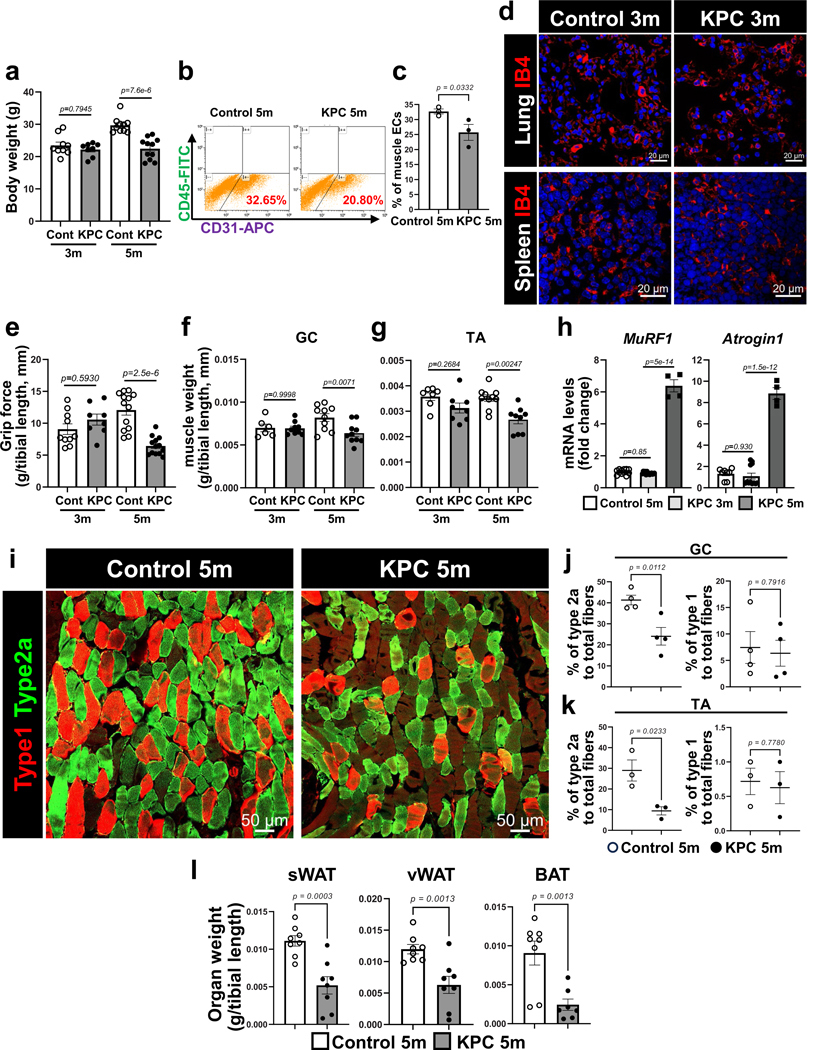
Cachexia phenotype in KPC mice. **a-e**. Characteristics of cachexia in control (3 and 5 month, referred to as 3 m and 5 m) and KPC (3 m and 5 m) mice. **a**. Body weight (n = 9 for control 3 m, n = 8 for KPC 3 m, n = 10 for control 5 m, n = 11 for KPC 5 m). Each dot represents one mouse. **b-c**. Representative flow plots of CD31 + CD45- muscle ECs (**b**) and quantification of muscle ECs by FACS analysis (**c**, n = 3). Each dot represents one mouse. **d**. The vasculature of the lung and spleen from age-matched control and KPC-3-month mice was evaluated by IB4 IF analysis. **e**. Four limbs grip strength of mice (n = 10 for control 3 m, n = 8 for KPC 3 m, n = 13 for control 5 m and KPC 5 m). Each dot represents one mouse. **f**. Gastrocnemius (GC) mass (n = 6 for control 3 m, n = 9 for KPC 3 m, n = 10 for control 5 m and KPC 5 m). Each dot represents one mouse. **g**. Tibialis anterior (TA) mass (n = 7 for control 3 m, n = 8 for KPC 3 m, n = 10 for control 5 m, n = 9 for KPC 5 m). Each dot represents one mouse. **h**. Expression of cachexia markers, *MuRF1* (n = 12 for control 5 m, n = 14 for KPC 3 m, n = 4 for KPC 5 m) and *Atrogin1* (n = 8 for control 5 m, n = 12 for KPC 3 m, n = 4 for KPC 5 m) in TA muscles by RT-qPCR. Each dot represents one mouse. **i-l**. Control-5month and KPC-5month mice were assessed for changes in muscle fiber types and fat mass. **i**. Representative images for type 2a and type 1 muscle fiber in GC muscles. **j-k**. Quantification of muscle fiber types in GC (**j**) (n = 4) and TA (**k**) muscles (n = 3). Each dot represents one mouse. **l**. The weight (g) of adipose tissues was normalized using tibial length (mm) (n = 8 for sWAT and vWAT, n = 8 for control BAT, n = 7 for KPC 5 m BAT). Each dot represents one mouse. **sWAT**: subcutaneous white adipose tissue, **vWAT**; visceral white adipose tissue, **BAT**; brown adipose tissue. Data are presented as mean ± SEM. Statistical analysis was conducted using unpaired, two-tailed t-test (**c, j, k, l**) or one-way ANOVA with Tukey’s multiple comparison test (**a, e-h**).

**Fig. 1 |:**
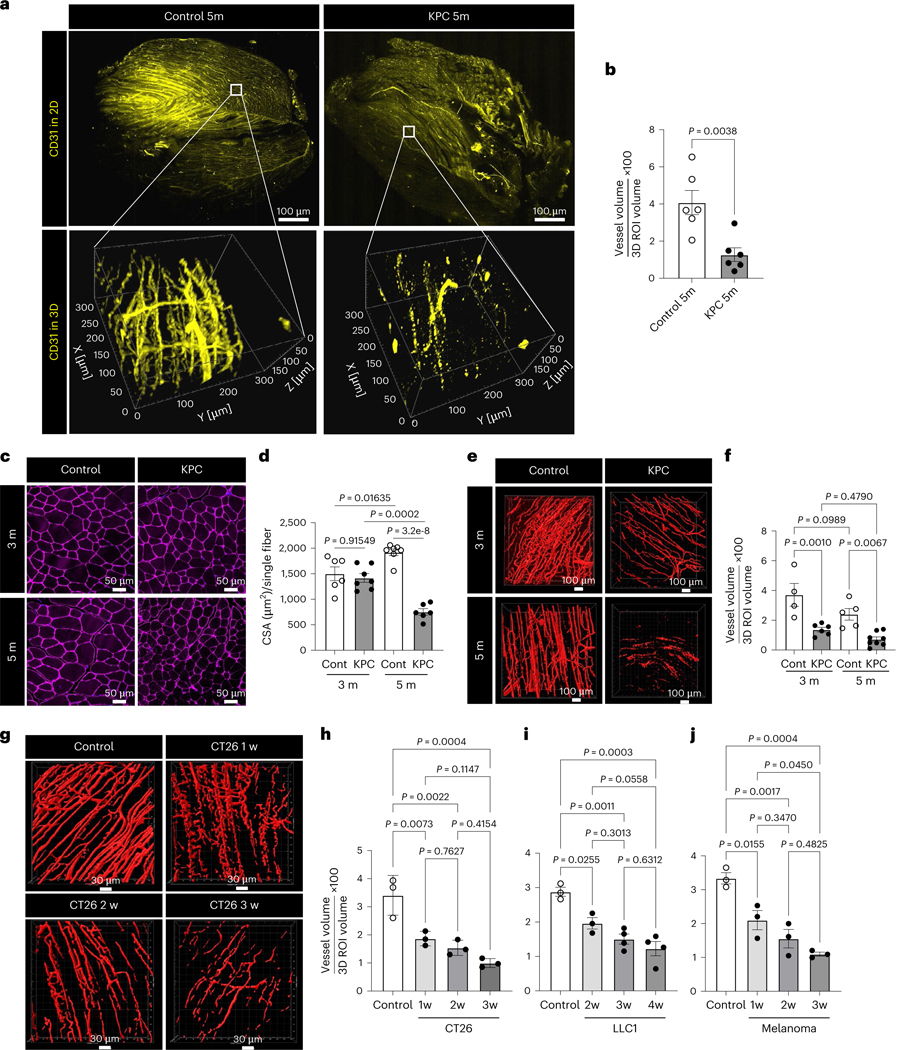
Cachexic muscles have reduced vascular density in experimental models of cancer. **a**, Representative images for high-resolution 3D muscle vasculature of the GC muscles from control 5-month-old and KPC 5-monthold mice. The optically cleared muscles were stained with CD31 antibody (pseudocolored yellow). **b**, Muscle vascular density (percentage of CD31^+^ vessel volume in 3D ROI volume; *n* = 6). Each dot represents one mouse. **c**–**f**, Comparison of control mice (3 and 5 months old) and KPC mice (3 and 5 months old). **c**, CSA of GC muscle based on immunofluorescence (IF) analysis with laminin (magenta) antibody. **d**, Quantification of CSA per single fiber (*n* = 6 for control 3-month-old and KPC 5-month-old mice and *n* = 7 for control 5-month-old and KPC 3-month-old mice). Each dot represents one mouse. **e**, Representative 3D images for surface volume of CD31^+^ (red) vessels. **f**, Quantification of muscle vasculature density (*n* = 4 for control 3-month-old mice, *n* = 6 for KPC 3-month-old mice, *n* = 5 for control 5-month-old mice and *n* = 8 for KPC 5-month-old mice). Each dot represents one mouse. **g**, Representative 3D images for the surface volume of CD31^+^ vessels of the GC muscles from CT26-bearing mice. **h**–**j**, Quantification of muscle vascular density for CT26 (**h**; *n* = 3), LLC1 (**i**; *n* = 3 for control and LLC1 at 2 weeks and *n* = 4 for LLC1 at 3 and 4 weeks) and B16F10 (**j**; *n* = 3). Each dot represents one mouse. Data are presented as the mean ± s.e.m. Statistical analysis was conducted using either an unpaired two-tailed *t*-test (**b**) or a one-way ANOVA with Tukey’s multiple-comparison test (**d**, **f** and **h**–**j**).

To explore the role of muscle vascular endothelial function in cancer cachexia, we used transgenic KPC (*LSL-Kras*^G12D/+^:*LSL-Trp53*^R172H/R172H^:*Pdx1-Cre*) mice^13,14^ that spontaneously develop fibrotic pancreatic adenocarcinoma and cachexia at approximately 4–6 months of age. The KPC 5-month-old mice had >10% lower body weight than age-matched control mice, whereas no such difference was seen at 3 months (Extended Data Fig. 1a). Thus, we refer to these 5-month-old KPC mice as KPC-cachexia mice. First, we evaluated muscle vascular density in intact muscle tissues using transparent tissue tomography^15^, which uses aqueous sugar solution-based immersion tissue clearing to enable high-resolution three-dimensional (3D) microscopy. Compared to the muscle capillary networks in control mice, which were intact and continuously connected, KPC-cachexia mice displayed substantially diminished and fragmented muscle vasculature (Fig. 1a) and decreased vascular surface volume (Fig. 1b). Muscle loss is primarily observed in the hindlimbs of cachexia mice^16^ and presents as reduced muscle fiber size in the tibialis anterior (TA), gastrocnemius (GC) and quadriceps in persons with cancer^17–19^. We observed that these muscles showed a significantly decreased percentage of ECs in the muscle of KPC-cachexia mice compared to control mice (Extended Data Fig. 1b,c).

To determine whether muscle mass correlated with muscle vascular density in cancer cachexia progression, we compared muscle size and vascular density in GC muscles from noncachexia KPC 3-month-old mice, cachexia KPC 5-month-old mice and age-matched control mice. The muscle cross-sectional area (CSA) was decreased in KPC 5-month-old mice compared to age-matched controls but this decrease was not apparent in KPC 3-month-old mice (Fig. 1c,d). Importantly, the vascular density of muscles was already reduced in the KPC 3-month-old mice and only modestly further decreased at 5 months (Fig. 1e,f), suggesting that the reduction in vascular density precedes muscle loss. No such reduction was observed in the vasculature of the lung or spleen (Extended Data Fig. 1d).

We examined whether loss of muscular vasculature in cancer cachexia affects functional performance and muscle mass by measuring mouse grip strength and muscle weight. We found a significant decrease in mouse grip strength and muscle mass in KPC 5-month-old mice but not KPC 3-month-old mice (Extended Data Fig. 1e–g). Importantly, the increased expression of cachexia markers, E3 ubiquitin ligases *MuRF1* and *Atrogin1* (ref. 20), was seen only in KPC 5-month-old mice but not KPC 3-month-old mice (Extended Data Fig. 1h). We characterized the nature of muscle loss by examining muscle fiber composition in KPC-cachexia mice compared to control mice. KPC 5-month-old mice showed a significant decrease in the proportion of type 2a fibers in GC and TA muscles but no change in type 1 fibers (Extended Data Fig. 1i–k). KPC 5-month-old mice also exhibited reduced adipose tissue mass (Extended Data Fig. 1l). These results suggest that the loss of muscle vascular density in KPC mice precedes the loss of muscle mass and function.

**Extended Data Fig. 2 |:**
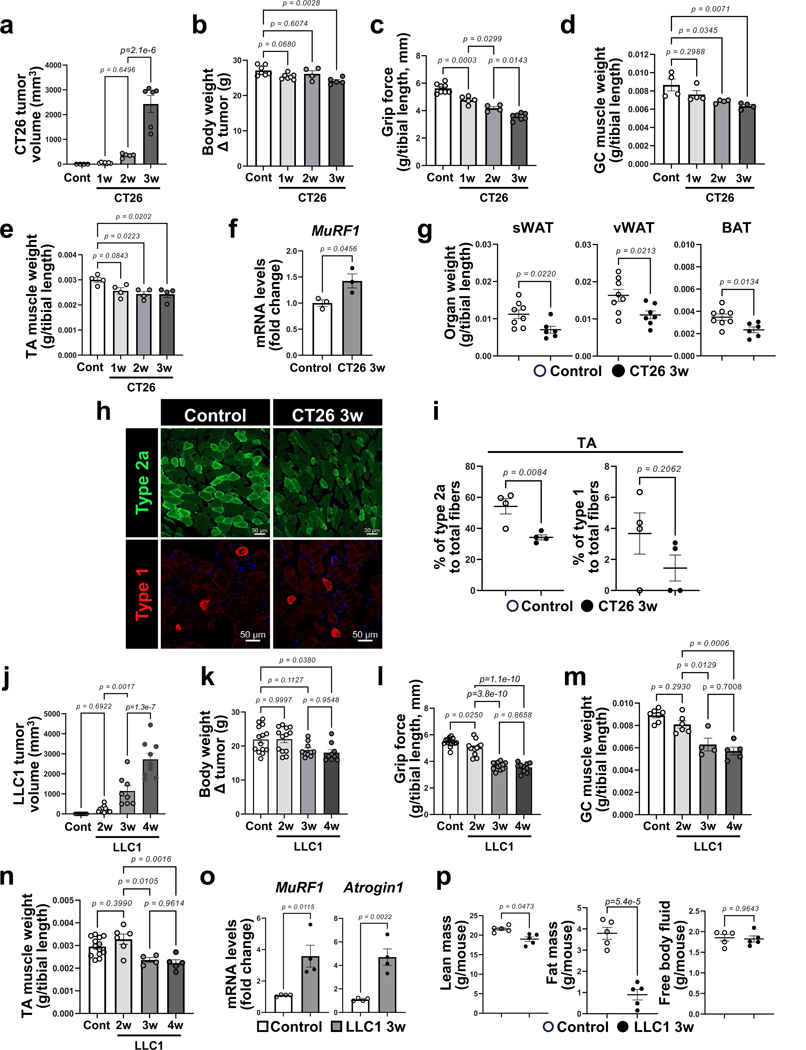
Cachexia phenotype in CT26 colon carcinoma- and LLC1 lung carcinoma-bearing mice. **a-i**. Control and CT26-bearing mice. **a**. Tumor growth post CT26 implantation (n = 4 for control, n = 7 for 1w, n = 5 for 2w, n = 6 for 3w). Each dot represents one mouse. **b**. Body weight was presented by subtracting (Δ) tumor weight (n = 7 for control, n = 7 for 1w, n = 4 for 2w, n = 5 for 3w). Each dot represents one mouse. **c**. Four limbs grip strength (n = 8 for control, n = 5 for 1w, n = 4 for 2w, n = 7 for 3w). Each dot represents one mouse. **d**. GC muscle mass (n = 4). Each dot represents one mouse. **e**. TA muscle mass (n = 4). Each dot represents one mouse. **f**. Expression of cachexia markers, *MuRF1* in TA muscles by RT-qPCR (n = 3). Each dot represents one mouse. **g**. The fat mass (g) normalized by tibial length (mm) (n = 8 for control, n = 6 for sWAT and BAT in CT26 3w, n = 7 for vWAT in CT26 3w). Each dot represents one mouse. **h**. Representative images for type 2a and type 1 muscle fiber in TA muscles. **i**. Quantification of muscle fiber types in TA muscles (n = 4). Each dot represents one mouse. **j-p**. Control and LLC1 bearing mice. **j**. Tumor growth post LLC1 implantation (n = 13 for control, n = 13 for 2w, n = 8 for 3w, n = 9 for 4w). Each dot represents one mouse. **k**. Body weight was presented by subtracting (Δ) tumor weight (n = 14 for control, n = 13 for 2w, n = 10 for 3w, n = 9 for 4w). Each dot represents one mouse. **l**. Four limbs’ grip strength (n = 14 for control, n = 12 for 2w, n = 12 for 3w, n = 10 for 4w). Each dot represents one mouse. **m**. GC muscle mass (n = 7 for control, n = 6 for 2w, n = 4 for 3w, n = 5 for 4w). Each dot represents one mouse. **n**. TA muscle mass (n = 13 for control, n = 6 for 2w, n = 4 for 3w, n = 5 for 4w). Each dot represents one mouse. **o**. Expression of cachexia markers, *MuRF1* and *Atrogin1* in TA muscles by RT-qPCR (n = 4). Each dot represents one mouse. **p**. Body composition of control and LLC1 3w mice by nuclear magnetic resonance (NMR) analysis (n = 5). Each dot represents one mouse. The lean, fat, or free body fluid mass was presented as absolute values per mouse without normalization. Data are presented as mean ± SEM. Statistical analysis was conducted using unpaired, two-tailed t-test (**f**, **g, i, o, p**) or one-way ANOVA with Tukey’s multiple comparison test (**a-e, j-n**).

**Extended Data Fig. 3 |:**
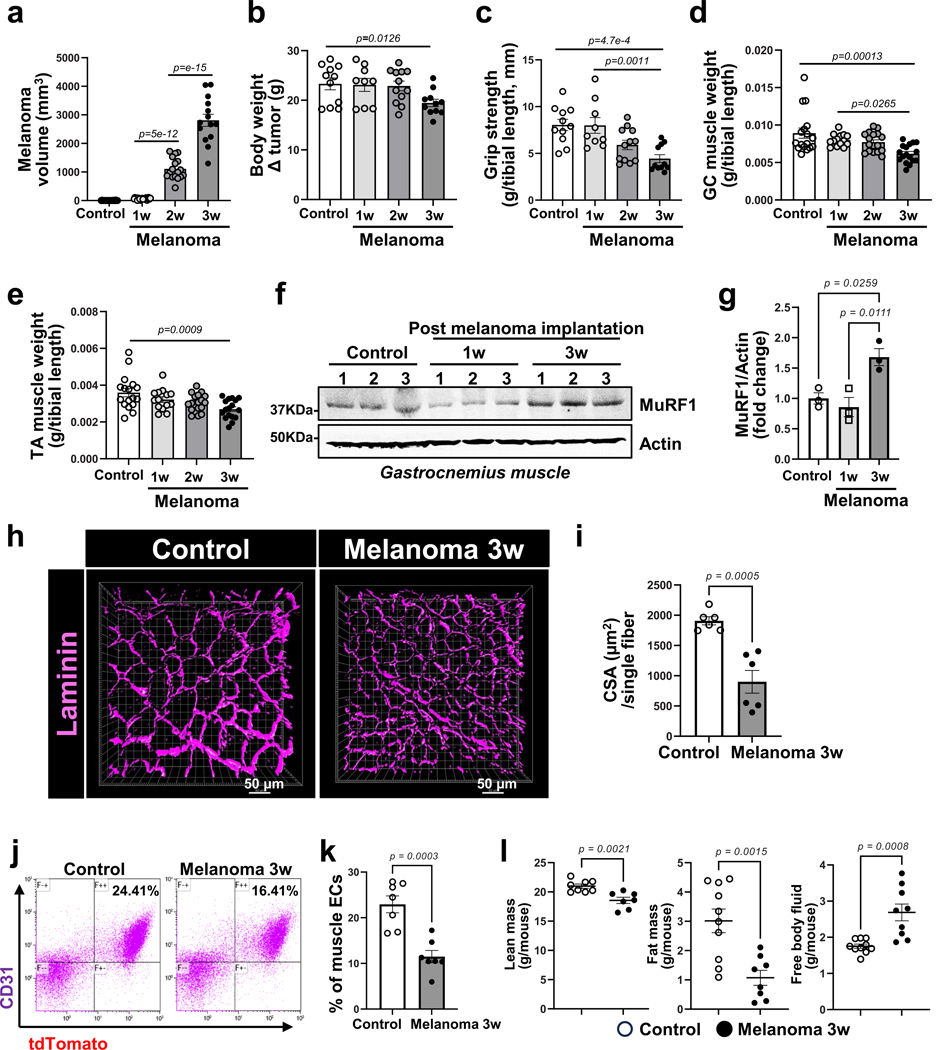
Cachexia phenotype in melanoma-bearing mice. **a**. Tumor growth post B16F10 implantation (n = 21 for control, n = 21 for 1w, n = 17 for 2w, n = 14 for 3w). Each dot represents one mouse. **b**. Body weight was presented by subtracting (Δ) tumor weight (n = 11 for control, n = 9 for 1w, n = 12 for 2w, n = 11 for 3w). Each dot represents one mouse. **c**. Four limbs’ grip strength (n = 11 for control, n = 9 for 1w, n = 12 for 2w, n = 11 for 3w). Each dot represents one mouse. **d-e**. Muscle mass for GC (**d**, n = 17 for control, n = 14 for 1w, n = 17 for 2w, n = 17 for 3w) and TA (**e**, n = 17 for control, n = 14 for 1w, n = 17 for 2w, n = 17 for 3w). Each dot represents one mouse. **f**. E3 ubiquitin ligase MuRF1 expression in GC muscles from control and melanoma-bearing mice (n = 3). Each lane represents one mouse. **g**. Quantification of MuRF1 protein levels in **f**. **h-l**. Comparison of control and melanoma 3-week mice. **h**. Cross-sectioned gastrocnemius muscle by IF analysis with a laminin antibody. The Z-sectioned images were reconstructed for visualization using Imaris software. **i**. Quantification of the CSA per single fiber (n = 6). Each dot represents one mouse. **j-k**. Representative flow plots of CD31+tdTomato+ muscle ECs (**j**) and quantification of muscle ECs by FACS analysis (**k**, n = 7). Each dot represents one mouse. **l**. Body composition of control and melanoma 3w mice by NMR analysis (lean mass; n = 8 for control, n = 7 for melanoma 3w, fat mass; n = 10 for control, n = 8 for melanoma 3w, free body fluid; n = 10 for control, n = 9 for melanoma 3w). Each dot represents one mouse. The lean, fat, or free body fluid mass was presented as absolute values per mouse without normalization. Data are presented as mean ± SEM. Statistical analysis was conducted using either unpaired, two-tailed t-test (**i, k, l**) or one-way ANOVA with Tukey’s multiple comparison test (**a-e**, **g**).

Next, we investigated whether our observations were specific to the KPC model or a more universal phenomenon in cancer cachexia. We expanded our study to include syngeneic allograft cancer models using CT26 colon carcinoma, Lewis lung carcinoma LLC1 and B16F10 skin melanoma^21^ cell lines. All three tumor models exhibited cachexia symptoms of reduced body weight, grip strength, muscle mass and type 2a fibers and increased expression of cachexia markers at 3–4 weeks after tumor implantation (Extended Data Fig. 2a–i for CT26, Extended Data Fig. 2j–p for LLC1 and Extended Data Fig. 3a–g for B16F10). Similar to the KPC model, a reduction in muscle vascular density preceded the development of cachexia in all three allograft models, beginning at 1–2 weeks after implantation (Fig. 1g–j). These models also showed a decrease in the mass of adipose tissues (Extended Data Fig. 2g,p), consistent with previous reports^22–26^. We further evaluated the cachexia phenotype in our melanoma model by quantifying muscle CSA (Extended Data Fig. 3h,i), muscle EC percentage (Extended Data Fig. 3j,k), lean mass and fat mass (Extended Data Fig. 3l).

**Extended Data Fig. 4 |:**
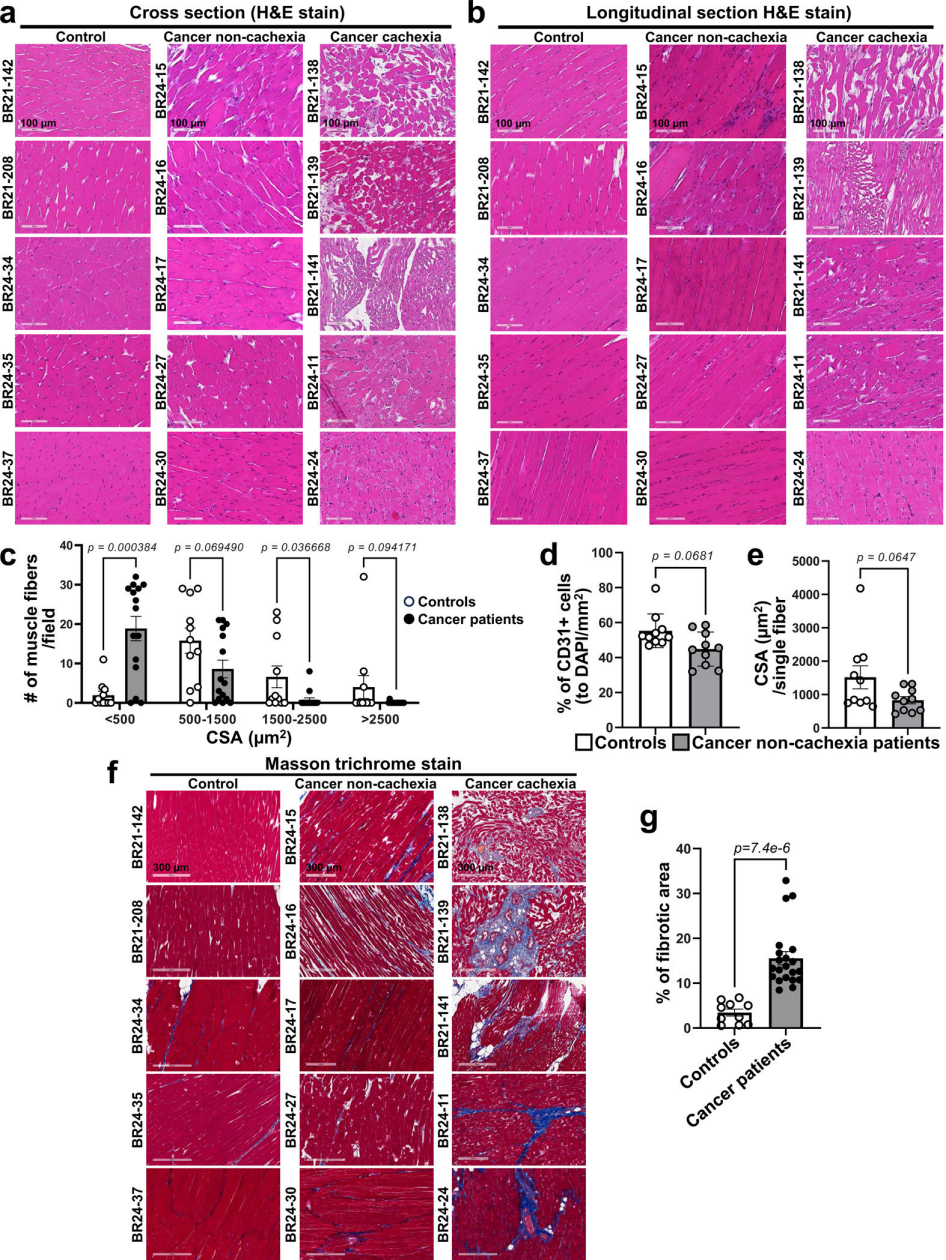
Cachexia phenotype in cancer patients. **a-g**. Evaluation of muscle structure and fibrosis from control subjects (n = 10) and cancer patients [non-cachexia (n = 11) *vs* cachexia (n = 10)]. Five representative H&E-stained sections were presented with cross-sectional areas (**a**) and longitudinal sectional areas (**b**). **c**. Size distribution of muscle fibers in control donors (n = 11) and cancer patients (n = 15). Each dot represents one patient. The statistical analysis was performed using a t-test for each size group, and BH p-value correction for all comparisons, and the adjusted p-values were reported. **d-e**. Comparison of muscle vascular density (**d**) or CSA (**e**) between controls (n = 10) and cancer non-cachexia patients (n = 10). Each dot represents one patient. **f**. Five representative images of muscle fibrosis by Masson’s trichrome staining. **g**. Quantification of muscle fibrosis area (n = 10 for controls, n = 21 for cancer patients). Each dot represents one patient. Statistical analysis was conducted using an unpaired, two-tailed t-test. Data are presented as mean ± SEM.

**Fig. 2 |:**
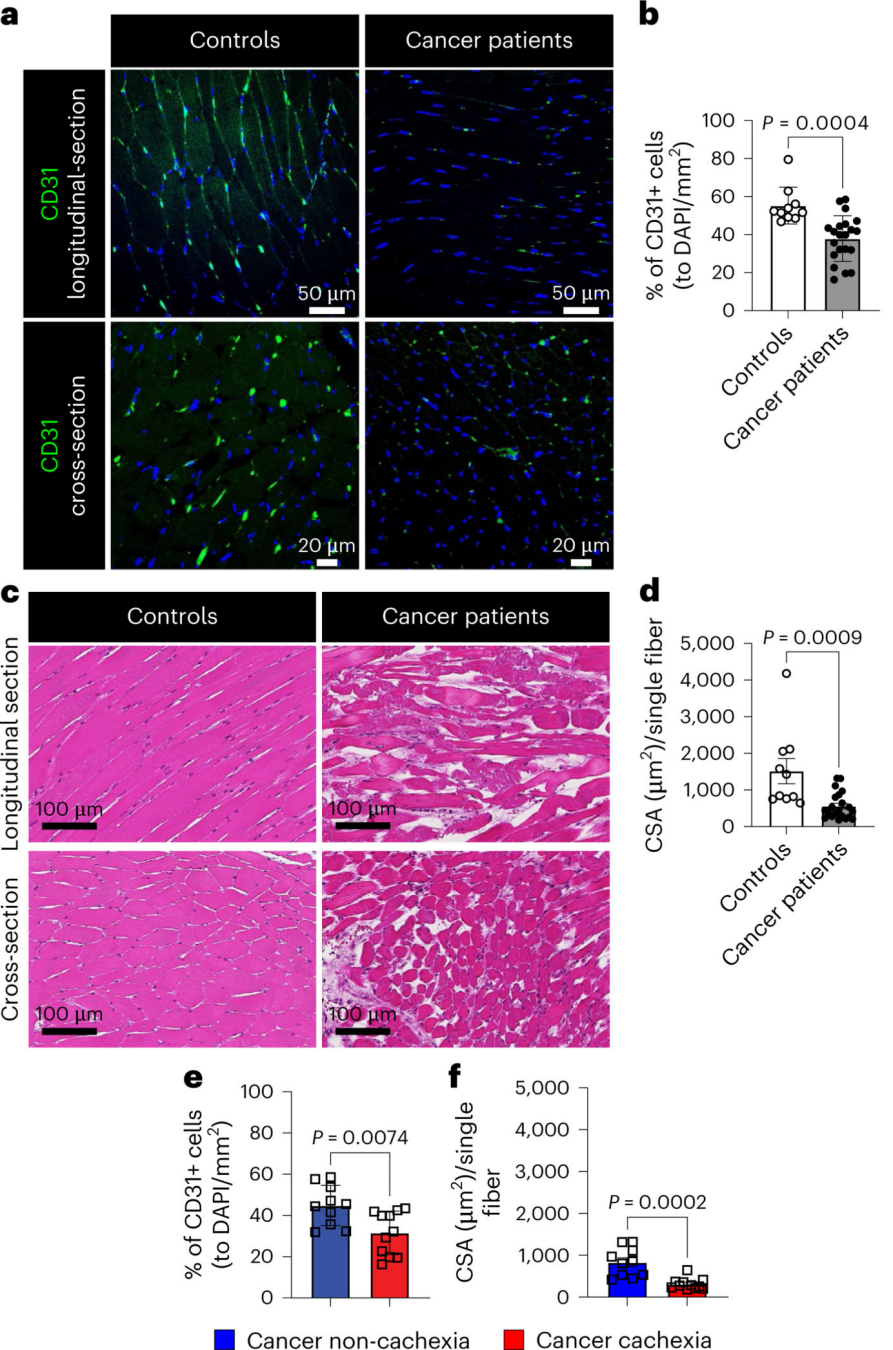
Participants with cancer cachexia show a decrease in muscle vascular density. **a**–**d**, Comparison of abdominal muscles from control subjects and participants with cancer. **a**, Muscle vasculature according to immunofluorescence (IF) analysis with CD31 (green) antibody and DAPI (blue) for the nucleus. **b**, Muscle vascular density (percentage of CD31^+^ cells as a function of total DAPI per area; *n* = 10 for controls and *n* = 21 for participants with cancer). Each dot represents one participant. **c**, H&E-stained muscle structure. **d**, CSA per single fiber (*n* = 10 for controls and *n* = 21 for participants with cancer). Each dot represents one participant. **e**,**f**, Comparison of muscle vascular density (**e**) or CSA (**f**) between participants with cancer without cachexia (*n* = 10) and those exhibiting cachexia (*n* = 11). Each dot represents one participant. Data are presented as the mean ± s.e.m. Statistical analysis was conducted using an unpaired two-tailed *t*-test (**b** and **d**–**f**).

To determine the translational relevance of muscle vascular rarefaction in cancer, we evaluated autopsied abdominal muscles from participants without cancer (referred to as controls) and participants with cancer. The muscles of participants with cancer showed markedly decreased muscle vascular density (Fig. 2a,b) and exhibited severe muscle atrophy (Fig. 2c,d and Extended Data Fig. 4a–c). We further analyzed the muscle vascular density in samples from participants with cancer meeting weight loss criteria for a cachexia diagnosis^27^ and in participants with cancer who did not meet cachexia criteria. Indeed, participants with cancer exhibiting cachexia had a significant reduction in both vascular density and CSA in muscle compared to those without overt cachexia (Fig. 2e,f). Importantly, participants with cancer without overt cachexia did not show a significant difference in muscle vascular density and muscle CSA when compared to controls (Extended Data Fig. 4d,e). We also observed that participants with cancer meeting cachexia criteria exhibited increased muscle fibrosis compared to controls (Extended Data Fig. 4f,g). These data underscore the translational relevance of skeletal muscle vascular loss in persons with cancer exhibiting cachexia.

### Dynamic differentially expressed genes in muscle ECs

**Extended Data Fig. 5 |:**
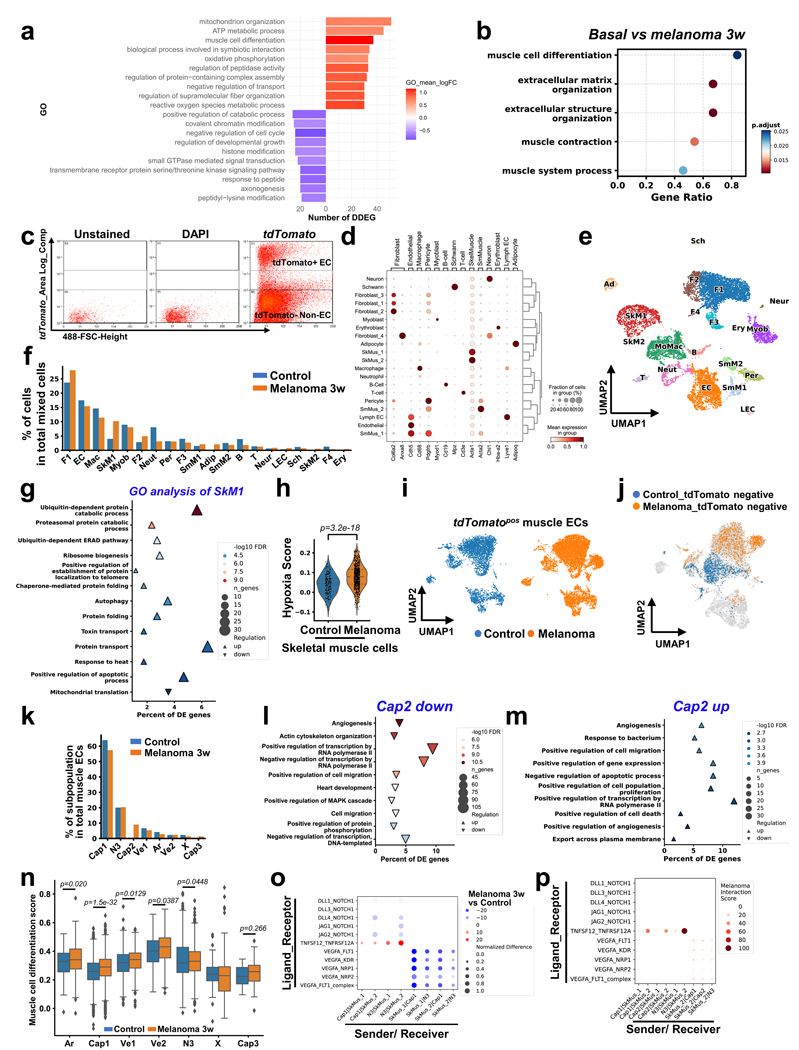
Unbiased transcriptomic analysis of muscle cells. **a-b**. Bulk RNA-seq analysis of dynamic differentially expressed genes (DDEG) in muscle vascular ECs. **a**. Top 20 GO pathways in muscle ECs enriched using accumulated log fold change DDEGs. **b**. Top 5 enriched GO terms using up-regulated DEGs from melanoma 3w compared to basal (0w) (n = 4). The ‘n’ represents the number of mice. **c-f**. scRNA-seq analysis of muscle cells and muscle ECs from control EC^tdTomato^ mice (n = 3) or melanoma 3w EC^tdTomato^ mice (n = 4). **c**. Gating strategy for tdTomato-positive muscle ECs or -negative muscle non-ECs by FACS sorting analysis for Fig. 2c–f. **d**. Cell-specific marker genes for total muscle tissue cells. **e**. Integrated UMAP for tdTomato-negative muscle cells. Mac; macrophage, F; fibroblast, Neut; neutrophil, Per; pericyte, Myob; myoblast, EC; endothelial cell, B; B cell, Sch; Schwann cell, T; T cell, SkM; skeletal muscle, SmM; smooth muscle cell, Neur; neuron, LEC; lymphatic ECs, Adip; adipocyte, Ery; erythroid-like. **f**. The relative proportion of specific cell types in total muscle cells. **g**. GO enrichment of differentially expressed genes in the SkM1 population. **h**. Hypoxia gene expression scores of individual skeletal muscle cells. The violin plots depict the kernel density estimate of the data which are shown as individual points. The horizontal lines depict the median and interquartile range. **i**. UMAPs for tdTomato positive (+) muscle ECs. **j**. Data from tdTomato negative ECs scRNA-seq analysis was integrated with tdTomato positive ECs scRNA-seq data set. tdTomato negative ECs (blue and orange) on tdTomato positive ECs (gray). **k**. The relative proportion of subpopulations in total muscle endothelial cells. Cap; capillary ECs, Ar; arterial ECs, Ve; vein ECs, N3; Notch3 expressing ECs, X; undefined. **l-m**. GO analyses of down-regulated (**l**) and up-regulated (**m**) Cap2 EC marker genes. **n**. The muscle cell differentiation module scores of individual cells were calculated based on genes differentially expressed in the bulk RNA-seq data. The box plots depict the median and interquartile range. The whiskers extend to the furthest data points within 1.5x the interquartile range beyond the box boundaries. Outliers beyond the whiskers are shown as individual points. **o-p**. Predicted ligand-receptor interactions between skeletal muscle cells and muscle ECs from melanoma-bearing mice relative to control mice (**o**) and within melanoma-bearing mice (**p**). The possible interactions in all muscle cell populations are available in Supplementary Tables 4, 5. Statistical analysis was conducted using an unpaired, two-tailed t-test (**h**, **n**).

**Fig. 3 |:**
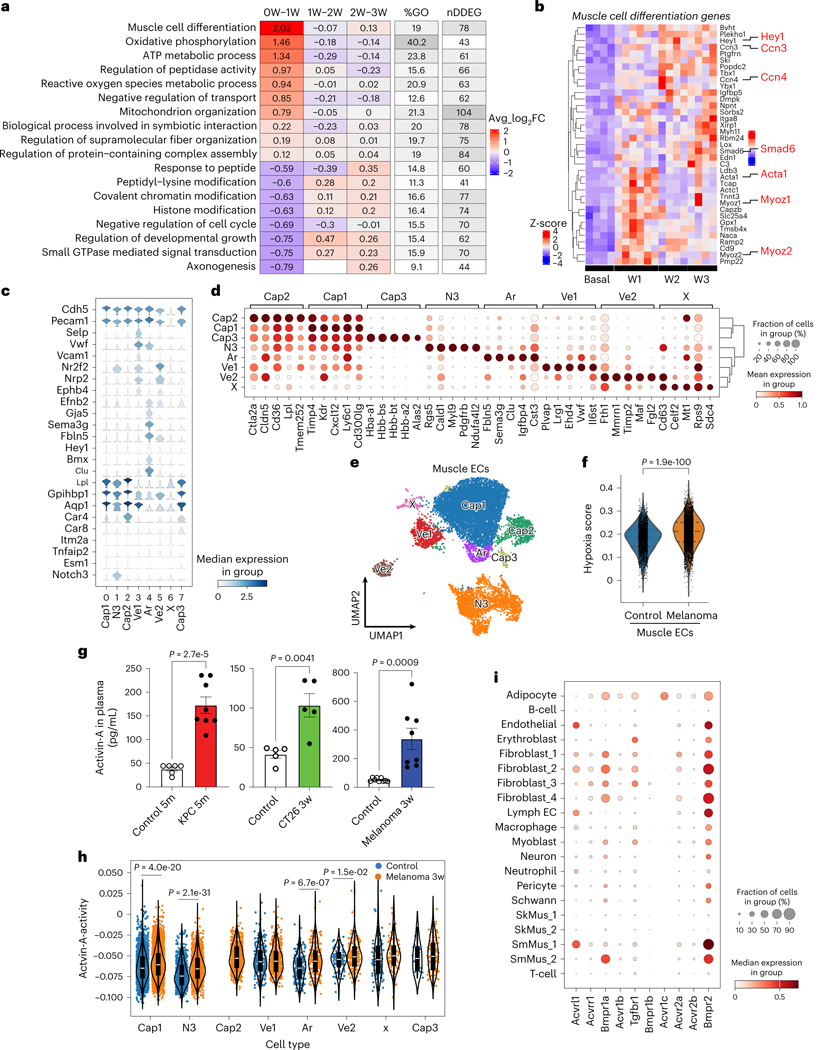
Expression of muscle cell differentiation-related genes and evaluation of activin A signaling in muscle endothelium. **a**,**b**, TrendCatcher analysis of DDEGs in muscle vascular ECs during tumor growth (control, *n* = 4 mice; week 1, *n* = 6 mice; week 2, *n* = 4 mice; week 3, *n* = 4 mice). **a**, TimeHeatmap of the top dynamic pathways. Each column represents a time interval. The number within each cell represents the averaged log_2_FC of gene expressions compared to the previous time point. The color represents the magnitude of the change. The ‘%GO’ column represents the percentage of DDEGs identified from the corresponding GO term. The ‘nDDEG’ column represents the number of DDEGs from the corresponding pathway. **b**, DDEG expression heat map from the biological pathway of muscle cell differentiation in **a**. Each column represents one mouse. The EndMT genes are highlighted. **c**–**i**, scRNA-seq analysis of muscle ECs from control EC^tdTomato^ mice (*n* = 3) or B16F10 3-week EC^tdTomato^ mice (*n* = 4). The gating strategy for FACS sorting is presented in Extended Data Fig. 5c. **c**, EC marker gene expression in muscle ECs. Cap, capillary; Ar, arterial; Ve, vein; N3, Notch 3 expressing; X, undefined. **d**, Top five unbiased marker genes in each muscle EC subpopulation. **e**, The integrated uniform manifold approximation and projection (UMAP) of tdTomato-positive muscle ECs. Each dot represents one cell. **f**, Hypoxia gene expression scores of individual ECs. The violin plots depict the kernel density estimate of the data, which are shown as individual points. The horizontal lines depict the median and interquartile range. **g**, The activin A levels in plasma (control 5-month-old mice, *n* = 6; KPC 5-month-old mice, *n* = 8; control PBS, *n* = 5; CT26 3-week mice, *n* = 5; control, *n* = 12; B16F10 3-week mice, *n* = 8). Each dot represents one mouse. **h**, Activin A signaling activity in muscle EC subpopulations (control, *n* = 3; B16F10 3-week mice, *n* = 4). The violins depict the kernel density estimate of the data, which are shown as individual points. The internal boxes represent the median and interquartile range. **i**, Expression of activin A receptors in all cell types. Data are presented as the mean ± s.e.m. Statistical analysis was conducted using an unpaired two-tailed *t*-test (**f**–**h**).

We next assessed the pathways underlying EC loss and remodeling throughout cachexia development using unbiased transcriptomic analyses. We used the melanoma model to study endothelial gene expression changes as it provides a robust and consistent time course of cachexia progression. We performed bulk RNA sequencing (RNA-seq) analysis on muscle ECs isolated from control and melanoma-bearing mice. We focused our analysis on dynamic differentially expressed genes (DDEGs) in muscle ECs during distal tumor growth using TrendCatcher^28^, which identifies genes with dynamic gene expression trajectories across multiple time points. We visualized global muscle endothelial transcriptomic changes using the TrendCatcher TimeHeatmap (Fig. 3a) and the top ten upregulated and downregulated biological pathways (Extended Data Fig. 5a and Supplementary Table 1). We found that the 78 DDEGs associated with muscle cell differentiation in ECs were modulated within the first week after tumor implantation. We performed a pairwise comparison between basal and 3-week melanoma using DESeq2 (ref. 29), which identified ‘muscle cell differentiation’ among the top five upregulated biological pathways in ECs (Extended Data Fig. 5b). Moreover, muscle ECs dynamically increased the expression of genes associated with energy metabolism and reactive oxygen species during distal tumor growth but decreased the expression of genes related to catabolic processes (Extended Data Fig. 5a). Several of the 78 DDEGs, including *Hey1*, *CCn3*, *CCn4*, *Smad6*, *Acta1*, *Myoz1* and *Myoz2* are related to endothelial-to-mesenchymal transition (EndMT)^30^ (Fig. 3b), suggesting that the surviving muscle ECs undergo a phenotypic shift during cachexia progression. Furthermore, these results support the hypothesis that tumors remotely regulate gene expression in muscle capillary ECs.

Single-cell transcriptomic analyses have demonstrated EC heterogeneity even within the same tissues^31,32^. Thus, we performed single-cell RNA-seq (scRNA-seq) analysis to examine muscle EC subpopulations using inducible EC-specific tdTomato lineage tracing mice (EC^tdTomato^). The tdTomato-positive cells (ECs) and tdTomato-negative cells (myocytes or other nonendothelial stromal cells) from the skeletal muscles of control EC^tdTomato^ and B16F10-cachexia EC^tdTomato^ mice (Extended Data Fig. 5c) were subjected to scRNA-seq. First, we performed a comparative analysis of all sequenced cells^33^. The cell types were assigned and clustered depending on cell-type-specific marker genes (Extended Data Fig. 5d,e and Supplementary Table 2). We found that the proportions of fibroblasts, skeletal muscle cells and adipocytes were increased in B16F10-cachexia mice when compared to control mice (Extended Data Fig. 5f). We analyzed DEGs in cluster SkM1, which was the predominant skeletal muscle cell population of B16F10-cachexia and control mice. The Gene Ontology (GO) enrichment analysis of the DEGs from this population showed significant increases in catabolic processes through activating pathways involving ubiquitination, proteasome function, autophagy, protein transport and apoptosis (Extended Data Fig. 5g), consistent with a cachexia phenotype. Skeletal muscle cells showed increased expression of hypoxia-response genes (GO:0071456) in B16F10-cachexia mice when compared to control mice (Extended Data Fig. 5h).

Next, we analyzed whether cachexic muscles have specific EC subpopulations associated with muscle loss. The muscle ECs clustered into eight subpopulations expressing specific muscle EC marker genes^34^ (Fig. 3c–e). Muscle ECs also showed significant increases in hypoxia gene expression in B16F10-cachexia mice compared to control mice (Fig. 3f). Interestingly, muscles of B16F10-cachexia mice demonstrated a unique Cap2 EC subpopulation not present in control mice (Extended Data Fig. 5i,k). Cap2 ECs showed decreased expression of angiogenesis genes (Extended Data Fig. 5l and Supplementary Table 3) but upregulated immune response and fat metabolism signatures (Extended Data Fig. 5m), suggesting that Cap2 ECs reduced angiogenic potential and EC functionality. We also found that multiple EC subpopulations from B16F10-cachexia mice expressed higher levels of muscle cell differentiation genes that we identified in our bulk RNA-seq analysis (Extended Data Fig. 5n), suggesting that several muscle EC subpopulations might be undergoing EndMT.

We hypothesized that circulating factors released by the tumor could drive the remodeling of the remote muscle vasculature. Activin A is a known tumor-derived secreted factor that is elevated in the blood, in blood vessels and within tumors^35–37^. Activin A promotes muscle wasting during cancer cachexia^37–42^ and suppresses EC proliferation under hypoxia^43^. We found that KPC-cachexia, CT26-cachexia and B16F10-cachexia mice exhibited higher levels of circulating activin A than control mice (Fig. 3g). Thus, we evaluated whether specific muscle EC subpopulations respond to circulating activin A by analyzing the score of activin A signaling activity as a function of the expression levels of its target genes^44^. We observed that activin A activity was higher in several muscle EC populations of tumor-bearing mice compared to control mice (Fig. 3h), correlating with the muscle differentiation score analysis in Extended Data Fig. 5n. Moreover, we analyzed the expression levels of activin A receptors in all muscle cell types using scRNA-seq data to compare sensitivity to activin A. While SkMus1 and SkMus2 cells had minimal expression levels of all activin A receptor subtypes, several muscle EC subpopulations markedly expressed activin A receptor-like type 1 (Fig. 3i), suggesting that the muscle endothelium is more susceptible to circulating activin A than skeletal muscle cells.

We next analyzed putative autocrine and paracrine signaling mediators between all muscle ECs and skeletal muscle cells on the basis of inferred ligand–receptor interactions using CellPhoneDB^45^. Predicted paracrine signaling pathways showed that skeletal muscle cells could interact with all muscle EC subpopulations through vascular endothelial growth factor (VEGF), Notch 1 and tumor necrosis factor (TNF) signaling pathways (Extended Data Fig. 5o,p and Supplementary Tables 4 and 5). These scRNA-seq data indicate that the emergence of the unique Cap2 EC subpopulation may serve as a sentinel population for muscle endothelial dysfunction in cancer cachexia. Importantly, there was a general upregulation of EndMT genes and activin A target genes across multiple muscle EC subpopulations.

### Dysfunctional ECs in cachexic muscles

**Extended Data Fig. 6 |:**
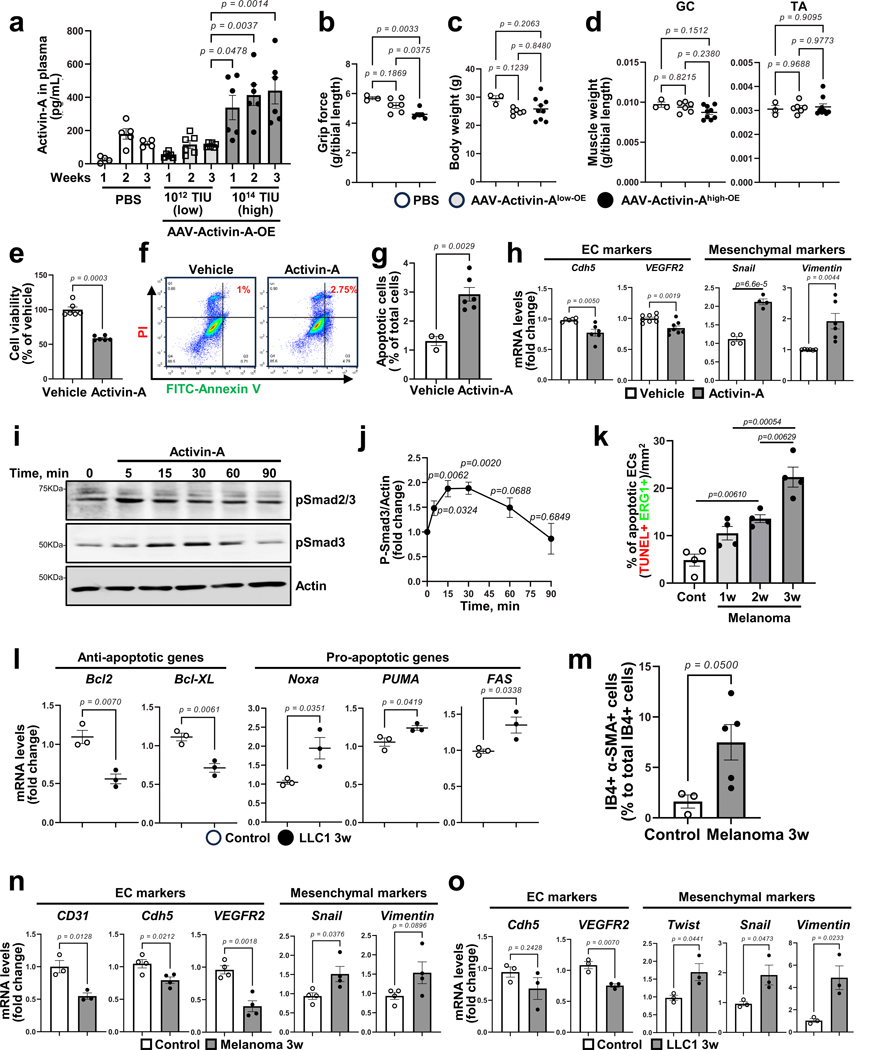
A decrease in muscle vascular density is associated with dysfunctional ECs in cachexic muscles. **a-d**. C57BL/6 WT mice were intravenously injected with AAV-Activin-A in a dose-dependent manner, and Activin-A levels were measured every week for 3 weeks. **a**. Activin-A levels in blood plasma following AAV-Activin-A overexpression (n = 4 for PBS 1w and 3w, n = 5 for PBS 2w, n = 6 for AAV-Activin-A^low-OE^ 1w, 2w, 3w, n = 6 for AAV-Activin-A^high-OE^ 1w, 2w, 3w). Each dot represents one mouse. **b**. Four limbs’ grip strength (n = 3 for PBS, n = 6 for AAV-Activin-A^low-OE^, n = 6 for AAV-Activin-A^high-OE^). Each dot represents one mouse. **c**. Body weight (n = 3 for PBS, n = 6 for AAV-Activin-A^low-OE^, n = 9 for AAV-Activin-A^high-OE^). Each dot represents one mouse. **d**. GC and TA muscle mass (n = 3 for PBS, n = 6 for AAV-Activin-A^low-OE^, n = 9 for AAV-Activin-A^high-OE^). Each dot represents one mouse. **e-g**. The HLMVECs were treated with Activin-A (25 ng/mL) for 24 h. **e**. HUVEC viability with and without Activin-A exposure (n = 6). Each dot represents one biological replicate. **f-g**. the apoptotic cells were evaluated with Annexin V-FITC + PI+ cells using FACS analysis. Representative flow plots (**f**) and quantification of apoptotic muscle ECs (**g**) (n = 3 for vehicle, n = 6 for Activin-A). Each dot represents one biological replicate. **h**. The expression of EndMT marker genes related genes by RT-qPCR (n = 6 for *Cdh5*, n = 8 for *VEGFR2*, n = 4 for *Snail*, n = 6 for *Vimentin*). Each dot represents one biological replicate. **i**. HLMVECs were treated with Activin-A (25 ng/mL) for the indicated time course (0, 5, 15, 30, 60, or 90 min) and followed by Western blotting. **j**. Quantification of the phospho-Smad3 levels in **i** (n = 3). Each dot represents one biological replicate. The statistical analysis was performed using a t-test with comparisons vs the 0 min time point. **k**. *In situ* TUNEL assay for apoptotic ECs in GC muscles of control (n = 4) and melanoma-bearing mice (n = 4). Double-positive cells of TUNEL and ERG1 in total ECs (ERG1 + ) were considered apoptotic ECs. Each dot represents one mouse. **l**. The expression of apoptosis genes in isolated muscle ECs from control (n = 3) and LLC1 cachexia (n = 3) mice by RT-qPCR. Each dot represents one mouse. **m**. Quantification of EndMT cells (double-positive cells of IB4 and α-SMA in total IB4+ ECs) in GC muscles of control and melanoma 3w mice (n = 3 for control, n = 5 for melanoma 3w). Each dot represents one mouse. **n**. The expression of EndMT marker genes in isolated muscle ECs from control and melanoma cachexia mice by RT-qPCR (n = 3 for *CD31*,n = 4 for *Cdh5*, *VEGFR2*, *Snail*, and *Vimentin*). Each dot represents one mouse. **o**. The expression of EndMT marker genes in isolated muscle ECs from control and LLC1 cachexia (n = 3) mice by RT-qPCR. Each dot represents one mouse. Data are presented as mean ± SEM. Each gene level was normalized by *18* *s* or *PPIA* levels and presented as a fold change. Statistical analysis was conducted using either unpaired, two-tailed t-test (**e**, **g**, **h**, **j**, **l**, **m**, **n**, **o**) or one-way ANOVA with Tukey’s multiple comparison test (**a-d**, **k**).

**Fig. 4 |:**
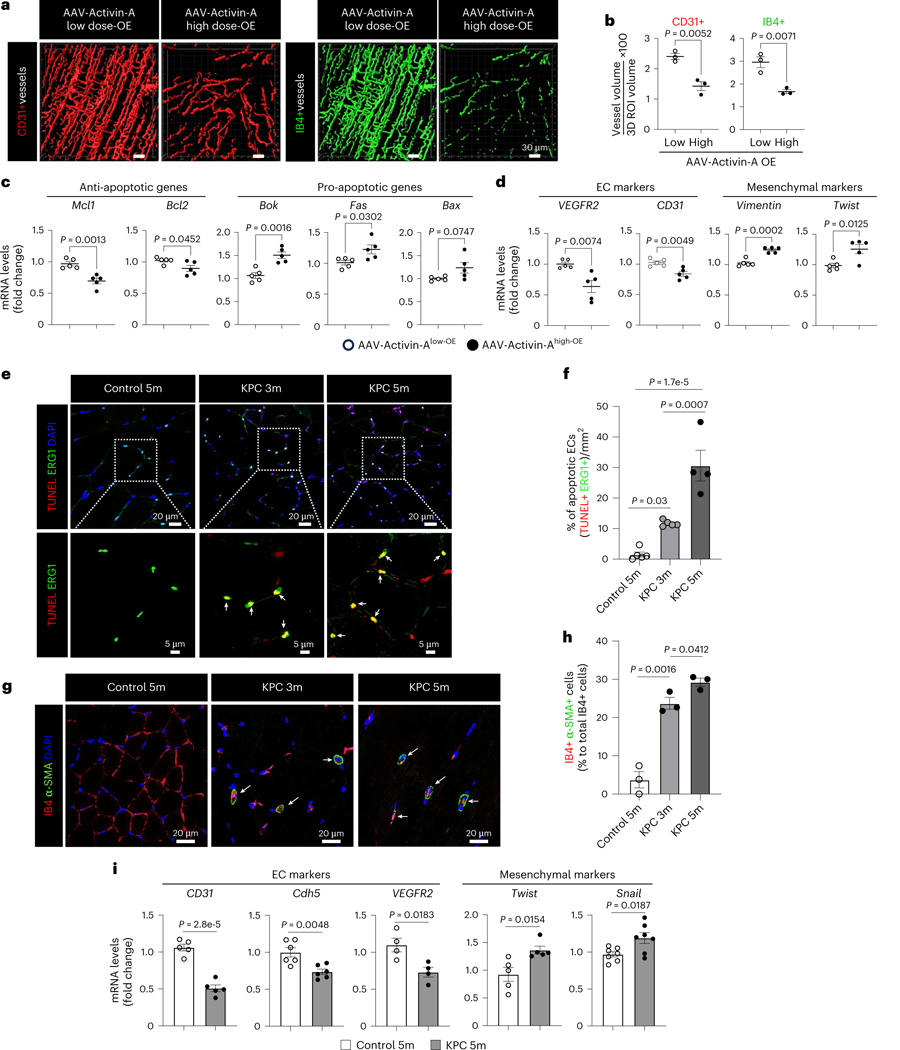
Increase in circulating activin A levels is associated with dysfunctional ECs in cancer cachexia progression. **a**–**d**, Control mice were intravenously injected with AAV-activin A low dose and AAV-activin A high dose and evaluated after 3 weeks. **a**, Representative 3D images for the surface volume of total CD31^+^ (red) or functional IB4^+^ (green) vessels in the GC muscles. **b**, Muscle vascular density (percentage of CD31^+^ or IB4^+^ vessel volume in 3D ROI volume; *n* = 3). Each dot represents one mouse. **c**,**d**, Muscle ECs isolated from mice overexpressing AAV-activin A low dose and AAV-activin A high dose were evaluated for the expression of apoptosis-related genes (**c**) or EndMT marker genes (**d**) by RT–qPCR (*n* = 5). Each dot represents one mouse. **e**–**h**, Control 5-month-old mice and KPC mice (3 and 5 months old). **e**, Cross-sectioned GC muscles were used for an in situ TUNEL assay for apoptotic cells and costained with endothelial TF EGR1 for ECs. Top, the selected areas (white square boxes) were enlarged to show the colocalization of TUNEL and EGR1. White arrows indicate the double-positive cells for TUNEL (red) and EGR1 (green) signal. **f**, Number of double-positive cells of TUNEL and EGR1 in **e** (*n* = 5 for control 5-month-old and KPC 3-month-old mice and *n* = 4 for KPC 5-month-old mice). Each dot represents one mouse. **g**, GC muscles were costained with IB4 for ECs and with anti-α-SMA antibody for mesenchymal cells. White arrows indicate the colocalization of IB4 (red) and α-SMA (green) signals. **h**, Quantification of IB4 and anti-α-SMA double-positive cells in **g** (*n* = 3). Each dot represents one mouse. **i**, Muscle ECs isolated from control 5-month-old and KPC 5-month-old mice were evaluated for the expression of EndMT marker genes by RT–qPCR (*n* = 5 for *CD31*, *n* = 6 for *Cdh5*, *n* = 4 for *VEGFR2*, *n* = 5 for *Twist* and *n* = 7 for *Snail*). Each dot represents one mouse. The nuclei were visualized with DAPI. Each gene level was normalized by *PPIA* levels and is presented as the FC. Data are presented as the mean ± s.e.m. Statistical analysis was conducted using an unpaired two-tailed *t*-test (**b**–**d** and **i**) or a one-way ANOVA with Tukey’s multiple-comparison test (**f** and **h**).

To determine whether elevated circulating activin A induces muscle endothelial dysfunction, we generated adeno-associated virus (AAV) activin A. Wild-type (WT) mice were intravenously injected with AAV-activin A in a dose-dependent manner and the circulating levels of activin A were monitored for 3 weeks (Extended Data Fig. 6a). We used two doses of AAV-activin A, a low dose mirroring levels of circulating activin A in saline-injected control mice and a high dose mirroring the levels seen in cancer cachexia mice. The high dose of AAV-activin A significantly reduced total CD31^+^ muscle vascular density and functional (perfused) isolectin B4 (IB4)^+^ vessels relative to the low dose (Fig. 4a,b). The high dose of AAV-activin A also significantly decreased mouse grip strength at 3 weeks after virus injection (Extended Data Fig. 6b) without affecting mouse body weight and muscle mass (Extended Data Fig. 6c,d). We next evaluated whether the loss of muscle vasculature was because of increased EC death or EndMT. The high dose of AAV-activin A significantly increased mRNA levels of proapoptosis and EndMT markers and concomitantly downregulated endothelial markers in muscle ECs when compared to the low dose (Fig. 4c,d). Ex vivo exposure of human microvascular ECs to activin A also significantly decreased cell viability (Extended Data Fig. 6e) but increased apoptosis, mRNA levels of EndMT markers and phosphorylated Smad3 proteins that regulate muscle cell differentiation (Extended Data Fig. 6f–j). These results suggest that activin A is a potent inducer of muscle endothelial dysfunction even in the absence of a tumor.

We next examined whether EC death was increased in cachexic muscles in vivo. The number of apoptotic ECs increased in the muscle vasculature even in KPC 3-month-old mice (Fig. 4e,f), which do not yet exhibit cachexia symptoms. Similarly, muscle EC apoptosis also preceded cachexia in melanoma-bearing mice (Extended Data Fig. 6k) and increased in LLC1-cachexia mice (Extended Data Fig. 6l). We also examined whether EndMT was induced in muscle ECs during cachexia progression by examining the colocalization of EC marker IB4 with mesenchymal marker α-smooth muscle actin (α-SMA). We observed that the percentage of IB4^+^α-SMA^+^ cells (EndMT cells) significantly increased in the muscles of KPC 3-month-old mice compared to control mice (Fig. 4g–h). Consistently, muscle ECs from B16F10-cachexia mice showed a higher percentage of IB4^+^α-SMA^+^ cells than control mice (Extended Data Fig. 6m). Moreover, isolated muscle ECs from cachexia mice showed significantly increased mRNA levels of EndMT markers relative to control mice (Fig. 4i for KPC, Extended Data Fig. 6n for B16F10 and Extended Data Fig. 6o for LLC1). Thus, circulating activin A induces both EC apoptosis and EndMT, contributing to the loss of functional muscle vasculature.

### Cachexic muscles exhibit vascular leakiness

**Extended Data Fig. 7 |:**
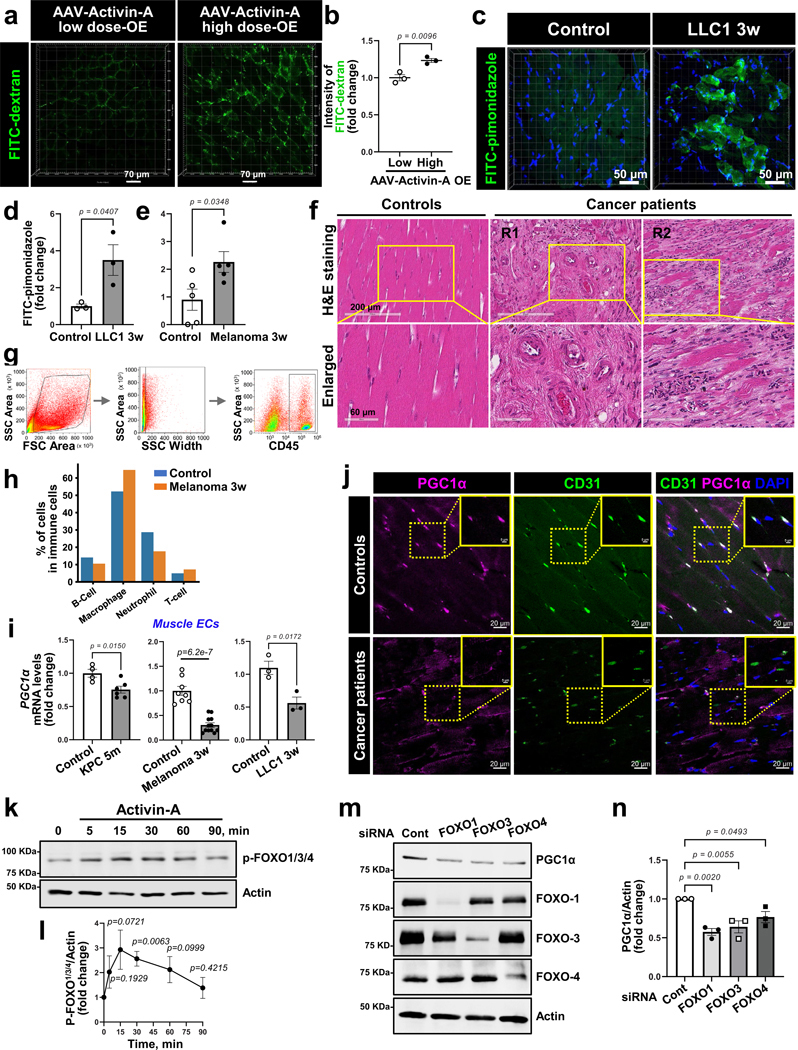
Decrease of muscle vascular density contributes to the increase of hypoxia and infiltration of immune cells in cachexic muscles. **a-b**. C57BL/6 WT mice were intravenously injected with AAV-Activin-A low dose and AAV-Activin-A high dose. After 3weeks, the mice were injected intravenously with IB4-A594 and fixable FITC-dextran before harvesting gastrocnemius muscles. **a**. Representative images of FITC-dextran+ (green) area. **b**. Intensity of FITC-dextran signal in **a** (n = 3). Each dot represents one mouse. **c-e**. The mice were intraperitoneally injected with pimonidazole (100 mg/kg) 1 h before harvesting muscle and the extent of hypoxia in gastrocnemius muscle was determined by the Hypoxyprobe Plus Kit. **c**. Muscle hypoxia in LLC1-cachexia mice. **d**. Quantification of surface volume of FITC-pimonidazole in **c** (n = 3). Each dot represents one mouse. **e**. Quantification of fluorescence intensity of FITC-pimonidazole in muscles of melanoma cachexia mice (n = 5). Each dot represents one mouse. **f**. H&E-stained abdominal muscles from control subjects and cancer patients. The yellow boxes were enlarged to show clear vessel structure and the infiltrated immune cells in near blood vessels. **R1** and **R2** in the upper panel indicate different regions from the same patient muscle. **g**. Gating strategy for flow cytometry analysis at Fig. 4g–h. **h**. The proportion (%) of immune cell type relative to total immune cells from control and melanoma 3w mice in the scRNA-Seq data. **i**. The *PGC1α* mRNA levels in isolated muscle ECs (n = 4 for control 5 m and n = 6 for KPC 5 m, n = 8 for control and n = 12 for melanoma 3w, n = 3 for control and LLC1 3w). Each dot represents one mouse. **j**. Expression of PGC1α in control and cancer patient muscle ECs by IF analysis (n = 5). The yellow boxes are enlarged to show the colocalization of CD31 and PGC1α. **k-l**. HLMVECs were treated with Activin-A (25 ng/mL) for indicated times. **k**. Immunoblotting to assess phospho-FOXO1/3/4 levels. **l**. Quantification of p-FOXO1/3/4 immunoblots shown in **k** (n = 3). Each dot represents one biological replicate. The statistical analysis was performed using a t-test with comparisons versus the 0 min time point. **m**. HLMVECs were transfected with specific siRNA for control, FOXO-1, −3, or −4. The protein levels of PGC1α, FOXO-1, −3, or −4 were determined by immunoblotting. **n**. Quantification of PGC1α levels in **m** (n = 3). Each dot represents one biological replicate. Data are presented as mean ± SEM. Statistical analysis was conducted using an unpaired, two-tailed t-test (**b**, **d, e, i, l**) or one-way ANOVA with Dunnett’s multiple comparison test (**n**).

**Fig. 5 |:**
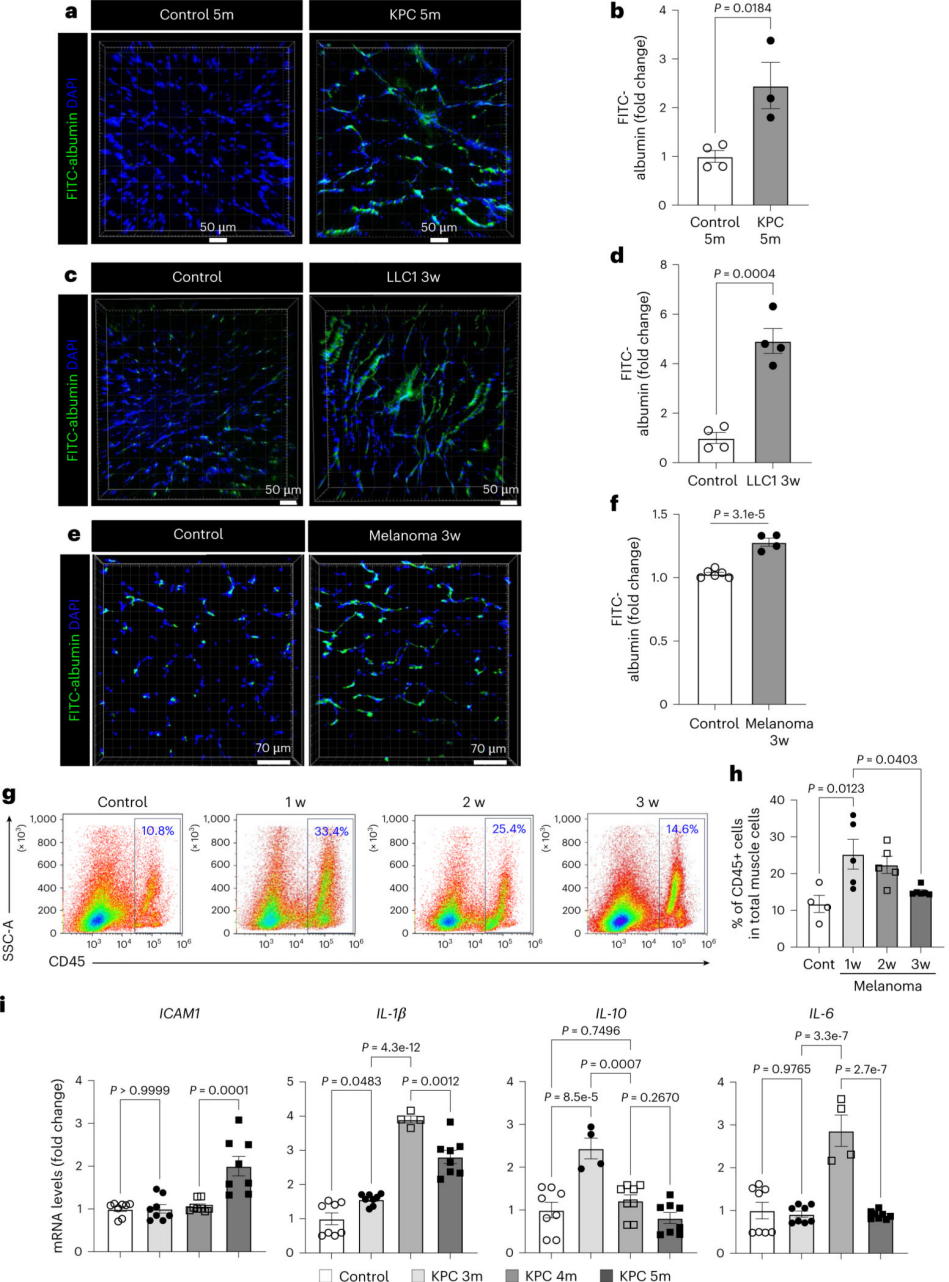
Cancer cachexic mice increase muscle vascular leakage and immune cell infiltration into muscles. **a**–**f**, The mice were retro-orbitally injected with FITC–albumin. Cryosections with 50-μm thickness of GC muscles were evaluated for vascular leakiness. **a**, Muscle vascular leakage in control 5-month-old and KPC 5-month-old mice. **b**, Quantification of surface volume of FITC–albumin in **a** (*n* = 4 for control 5-month-old mice and *n* = 3 for KPC 5-month-old mice). Each dot represents one mouse. **c**, Muscle vascular leakage in control and LLC1 3-week mice. **d**, Quantification of surface volume of FITC–albumin in **c** (*n* = 4). Each dot represents one mouse. **e**, Muscle vascular leakage in B16F10-cachexia mice. **f**, Intensity of FITC–albumin in **e** (*n* = 6 for control mice and *n* = 4 for B16F10 3-week mice). Each dot represents one mouse. **g**, The inflammatory cells were represented by percentage of CD45^+^ cells in whole muscle cells by FACS analysis. The gating strategy is presented in Extended Data Fig. 7g. **h**, Quantification of CD45^+^ cells (*n* = 4 for control and *n* = 5 for 1-week, 2-week and 3-week mice). Each dot represents one mouse. **i**, Inflammatory genes in TA muscles from control 5-month-old and KPC mice (3, 4 and 5 months old) by RT–qPCR (*ICAM1*, *n* = 8 for all groups; *IL1β*, *n* = 8 for control, KPC 3-month-old and KPC 5-month-old mice and *n* = 4 for KPC 4-month-old mice; *IL10*, *n* = 8 for control, KPC 4-month-old and KPC 5-month-old mice and *n* = 4 for KPC 3-month-old mice; *IL6*, *n* = 8 for control, KPC 3-month-old and KPC 5-month-old mice and *n* = 4 for KPC 4-month-old mice). Each dot represents one mouse. Each gene level was normalized by *PPIA* levels and is presented as the FC. Data are presented as the mean ± s.e.m. The *Z*-sectioned images were reconstructed for visualization using Imaris software and nuclei were stained with DAPI (**a**, **c** and **e**). Statistical analysis was conducted using an unpaired two-tailed t-test (**b**, **d** and **f**) or a one-way ANOVA with Tukey’s multiple-comparison test (**h** and **i**).

We assessed endothelial barrier integrity as a key readout of intact vascular function in cancer cachexia mice. Interestingly, the injection of a high dose of AAV-activin A in WT mice significantly increased the microvascular leakage compared to the low dose (Extended Data Fig. 7a,b). The KPC-cachexia, LLC1-cachexia and B16F10-cachexia mice also showed substantially higher microvascular leakiness in the GC muscles when compared to control mice (Fig. 5a–f). Moreover, the muscles of LLC1-cachexia and B16F10-cachexia mice showed significantly increased hypoxia (Extended Data Fig. 7c–e), indicative of reduced perfusion in the rarified and leaky muscle vasculature.

Next, we examined whether the impairment of the endothelial barrier could also promote immune cell infiltration. As shown in Extended Data Fig. 7f, the muscles of participants with cancer showed increased immune cell infiltration near capillaries and interstitial spaces. In melanoma-bearing mice, the total number of CD45^+^ immune cells in muscles significantly surged as early as week 1 and then gradually decreased over tumor progression (Fig. 5g,h and Extended Data Fig. 7g). Our scRNA-seq data also showed an increase in the relative proportion of macrophages within total immune cells (Extended Data Fig. 7h). Furthermore, we found that the muscles of KPC mice had increased expression of inflammatory mediators during cachexia progression (Fig. 5i).

### EC peroxisome proliferator-activated receptor γ coactivator 1α is required for muscle homeostasis

**Fig. 6 |:**
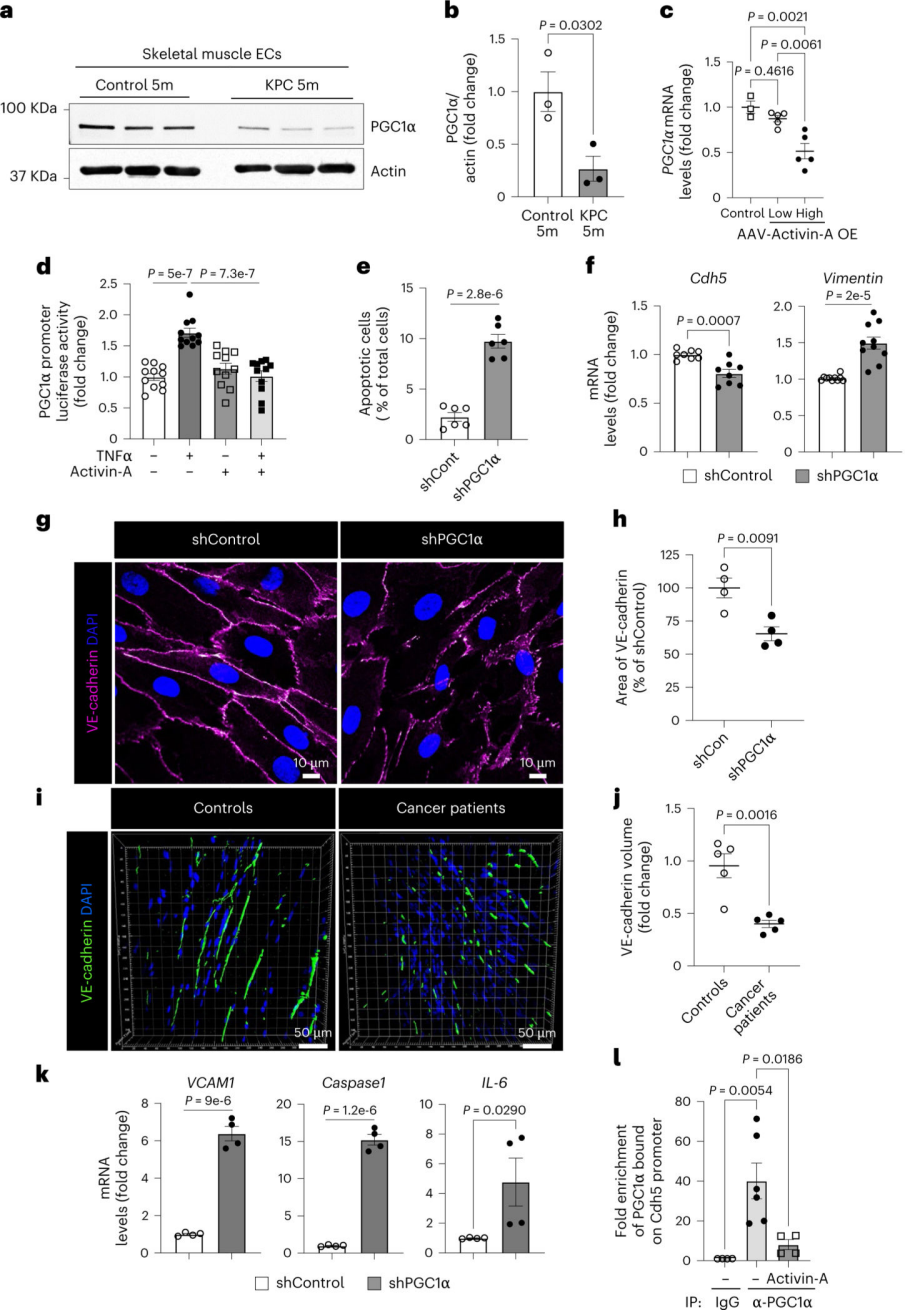
Circulating activin A induces vascular dysfunction by suppressing endothelial PGC1α in muscle. **a**, PGC1α protein levels in isolated muscle ECs from control 5-month-old and KPC 5-month-old mice by western blotting. Each lane represents one mouse. **b**, Quantification of PGC1α in **a** (*n* = 3). Each dot represents one mouse. **c**, *PGC1α* mRNA levels in isolated muscle ECs. The mice were evaluated 3 weeks after injecting AAV-control, AAV-activin A low dose or AAV-activin A high dose (*n* = 3 for control and *n* = 5 for AAV-activin A low and high doses). Each dot represents one mouse. **d**, HLMVECs were transfected with PGC1α promoter luciferase plasmid and *Renilla* luciferase for 48 h and treated with vehicle (0.1% BSA), TNF (10 ng ml^−1^), activin A (25 ng ml^−1^) or a combination of TNF and activin A for 16 h. The luciferase activity was normalized by *Renilla* luciferase and is presented as the FC (*n* = 11). Each dot represents one independent biological replicate. **e**–**h**, HLMVECs were treated with lentiviral shRNA for control and PGC1α for 72 h. **e**, The apoptotic cells (annexin V–FITC^+^PI^+^) were evaluated by FACS analysis (*n* = 6). Each dot represents one independent biological replicate. **f**, Levels of EndMT marker genes, *Cdh5* and *Vimentin*, by RT–qPCR (*n* = 8). Each dot represents one independent biological replicate. **g**, EC barrier integrity by IF staining with VE-cadherin antibody. Nuclei were stained with DAPI. **h**, Quantification of the area of VE-cadherin in **g** (*n* = 4). Each dot represents one independent biological replicate. **i**,**j**, Muscle vascular barrier integrity of controls and participants with cancer by IF staining with VE-cadherin antibody. **i**, Representative VE-cadherin images. **j**, The surface volume of VE-cadherin (*n* = 5). Each dot represents one participant. **k**, Levels of inflammatory genes by RT–qPCR (*n* = 4). Each dot represents one independent biological replicate. **l**, ChIP analysis in control and activin A-treated ECs with PGC1α-specific antibody. Normal mouse IgG antibody was used as a negative control. The enrichment values were normalized with input values and are presented as the FC (*n* = 4 for control cells for IgG IP, *n* = 6 for control cells for anti-PGC1α IP and *n* = 4 for activin A-treated cells for anti-PGC1α IP). Each dot represents one independent biological replicate. Data are presented as the mean ± s.e.m. Statistical analysis was conducted using either an unpaired two-tailed t-test (**b**, **e**, **f**, **h**, **j** and **k**) or a one-way ANOVA with Tukey’s multiple-comparison test (**c**, **d** and **l**).

We next examined whether transcription factors (TFs) were differentially active in the muscle ECs of B16F10-cachexia mice by analyzing the promoter regions of DEGs for binding motif enrichment. We observed multiple enriched binding motifs for downregulated genes in muscle ECs of B16F10-cachexia mice (Supplementary Table 6). The most significant enrichment was for KLF15, a key TF involved in endothelial homeostasis^46^. Interestingly, the downregulated genes in muscle ECs of B16F10-cachexia mice were enriched for multiple cofactors of the transcriptional coactivator peroxisome proliferator-activated receptor (PPAR)γ coactivator 1α (PGC1α), such as Nrf1, PPARδ, Mef2, Hnf4 and forkhead box O1 (FOXO1). Given that PGC1α supports cell survival in many cell types by regulating energy and redox signaling^47^, we hypothesized that EC PGC1α may be downregulated in cachexia and this downregulation could mediate the observed vascular dysfunction. The muscle ECs of KPC-cachexia mice indeed showed significantly lower protein levels of PGC1α compared to control mice (Fig. 6a,b). The mRNA levels of PGC1α were also significantly decreased in isolated muscle ECs in KPC-cachexia, LLC1-cachexia and B16F10-cachexia mice compared to control mice (Extended Data Fig. 7i). Importantly, participants with cancer exhibiting cachexia also showed a decrease in PGC1α protein levels in muscle ECs compared to controls (Extended Data Fig. 7j).

Next, we examined whether activin A affects PGC1α expression in muscle ECs even in the absence of tumors. The high dose of AAV-activin A significantly decreased *PGC1α* mRNA levels in muscle ECs compared to the low dose (Fig. 6c). Mechanistically, activin A exposure decreased the expression of PGC1α in TNF-activated human lung microvascular ECs (HLMVECs) by suppressing PGC1α promoter activity (Fig. 6d). To identify TFs associated with the activin A signaling pathway, we examined the phosphorylation of the FOXO family, which inhibits its nuclear translocation^48^. Activin A exposure significantly increased phosphorylation of FOXO1 (T^24^), FOXO3 (T^32^) and FOXO4 (T^28^) (Extended Data Fig. 7k–l). Moreover, the depletion of FOXO1, FOXO3 or FOXO4 significantly decreased the expression levels of PGC1α in ECs (Extended Data Fig. 7m,n). These results suggest that activin A inhibits the binding of FOXO TFs at the PGC1α promoter and downregulates PGC1α expression in ECs.

**Extended Data Fig. 8 |:**
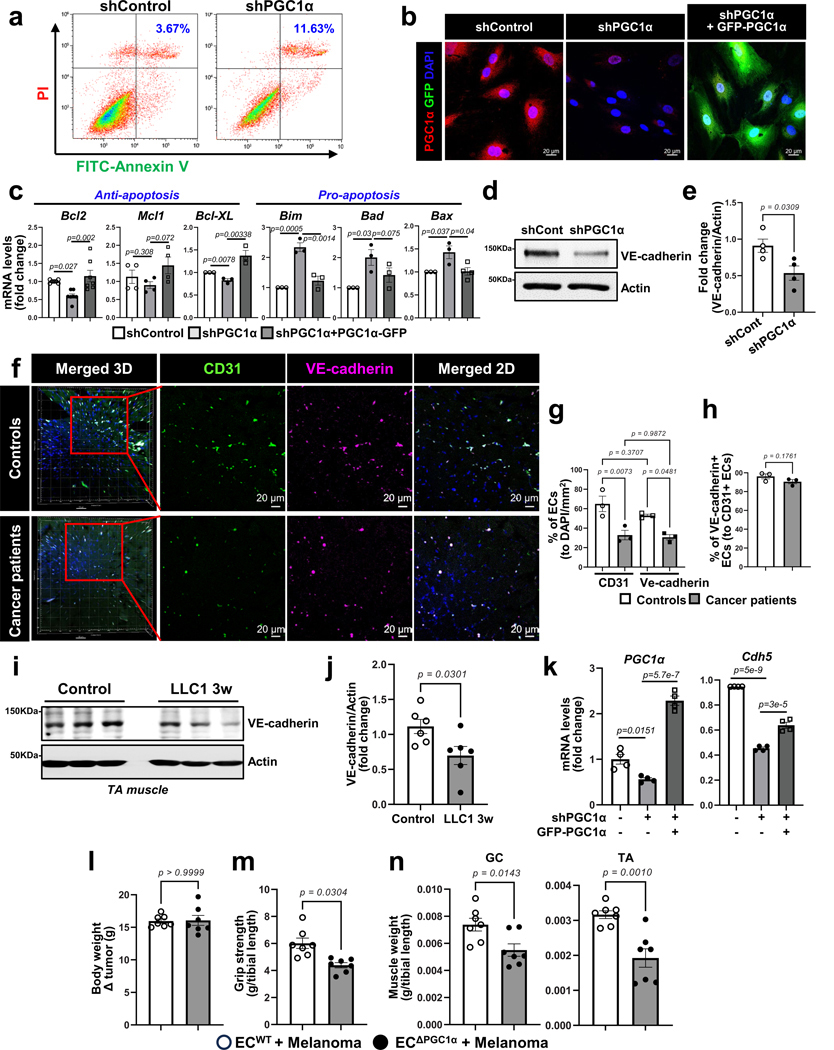
Endothelial PGC1α is required for endothelial barrier integrity. **a-e**. HLMVECs were transfected with lentiviral shRNA for PGC1α without or with GFP-PGC1α plasmid. **a**. Representative flow plots of apoptotic cells. **b**. PGC1α expression levels by IF analysis (n = 3). The red and green colors represent the endogenous PGC1α (red) and exogenous GFP-PGC1α (green), respectively. **c**. Apoptotic genes (n = 7 for *Bcl2*, n = 4 for *Mcl1*, n = 3 for *Bcl-XL, Bim and Bad, Bax:* n = 3 for shControl and shPGC1α, n = 4 for shPGC1α + PGC1α-GFP). Each dot represents one biological replicate. **d**. The protein levels of VE-cadherin. **e**. Quantification of VE-cadherin in **d** (n = 4). Each dot represents one biological replicate. **f-h**. Abdominal muscles from control subjects and cancer patients. **f**. Representative images for either CD31+ or VE-cadherin+ ECs by IF analysis. **g**. Quantification of either CD31+ or VE-cadherin+ ECs in **f** (n = 3). Each dot represents one patient. **h**. Percent of VE-cadherin+ ECs to CD31+ ECs (n = 3). Each dot represents one patient. **i**. VE-cadherin protein levels in TA muscles from control and LLC1-cachexia mice (n = 3). Each lane represents one mouse. **j**. Quantification of the levels of VE-cadherin in **i** (n = 6). Each dot represents one mouse. **k**. *PGC1α* and *Cdh5* mRNA levels in PGC1α depleted HLMVECs with or without GFP-PGC1α overexpression (n = 4). Each dot represents one biological replicate. **l-n**. Characterization of melanoma 3w-bearing EC^WT^ and ECΔ^PGC1α^ mice. **l**. Body weight (n = 7). Each dot represents one mouse. **m**. Grip strength (n = 7). Each dot represents one mouse. **n**. Muscle weight (n = 7). Each dot represents one mouse. Data are presented as mean ± SEM. Each gene level was evaluated by RTqPCR, normalized by *18* *s* levels and presented as fold change. Statistical analysis was conducted using unpaired two-tailed t-test (**e, h**, **j, l-n**), or one-way ANOVA with Tukey’s multiple comparison test (**c**, **g, k**).

Next, we evaluated whether loss of EC PGC1α phenocopies the effects of activin A on endothelial function. PGC1α-depleted HLMVECs showed a significant increase in apoptosis relative to control cells (Fig. 6e and Extended Data Fig. 8a). Conversely, the overexpression of GFP–PGC1α in PGC1α-depleted HLMVECs restored antiapoptotic gene expression and inhibited proapoptotic gene expression (Extended Data Fig. 8b,c). Furthermore, EC PGC1α depletion significantly decreased mRNA levels of EC marker *Cdh5* (Fig. 6f, left) but also increased expression levels of the mesenchymal marker *Vimentin* (Fig. 6f, right), indicating that PGC1α downregulation may be inducing EndMT.

The endothelial adherens junctional protein VE-cadherin is critical for maintaining an intact vascular barrier. However, PGC1α-depleted ECs had markedly disrupted endothelial adherens junctions as a result of reduced VE-cadherin levels (Fig. 6g–h and Extended Data Fig. 8d,e). Muscle samples of participants with cancer also demonstrated significantly reduced VE-cadherin when compared to those of controls (Fig. 6i,j) and this decrease was accompanied by a decrease in overall muscle vascular density as assessed by staining for the endothelial marker CD31 (Extended Data Fig. 8f–h). Moreover, LLC1-cachexia mice demonstrated significantly decreased VE-cadherin in TA muscles when compared to controls (Extended Data Fig. 8i–j). Conversely, the overexpression of EC PGC1α in PGC1α- depleted ECs restored the expression of VE-cadherin mRNA (*Cdh5)* levels (Extended Data Fig. 8k). Additionally, the PGC1α-depleted ECs had significantly increased mRNA levels of proinflammatory genes compared to control cells (Fig. 6k). We then assessed how PGC1α regulates VE-cadherin expression in ECs. Because the human VE-cadherin promoter has two putative PPARγ-binding motifs to which PGC1α binds as a transcriptional coactivator, we designed primers to recognize the PPARγ-binding motifs to determine whether PGC1α can indirectly bind PPARγ-binding motifs on the VE-cadherin promoter. In a chromatin immunoprecipitation (ChIP) assay, PGC1α antibody strongly bound the VE-cadherin promoter in control ECs but its binding was significantly inhibited by activin A treatment (Fig. 6l).

**Fig. 7 |:**
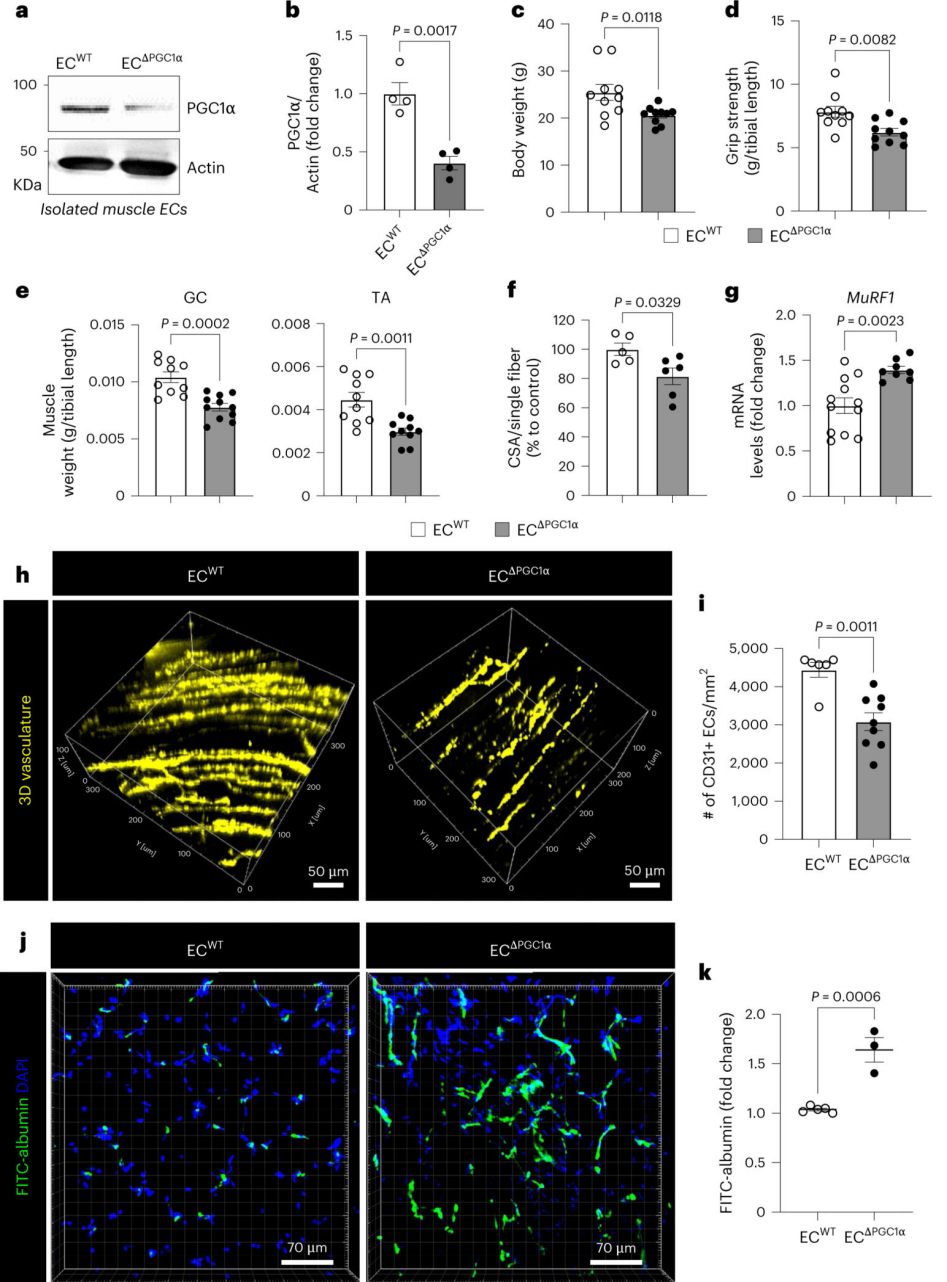
Endothelial-specific PGC1α depleted mice manifest elements of cachexia phenotypes. **a**–**k**, EC^WT^ (EC^tdTomato,WT^) and EC^ΔPGC1α^ (EC^tdTomato,ΔPGC1α^) mice. **a**, PGC1α knockdown efficiency in isolated muscle ECs. **b**, Quantification of PGC1α levels in **a** (*n* = 4). Each dot represents one mouse. **c**, Body weight (*n* = 10). Each dot represents one mouse. **d**, Grip strength (*n* = 10). Each dot represents one mouse. **e**, Muscle weight (*n* = 10). Each dot represents one mouse. **f**, CSA per single fiber (*n* = 5 for EC^WT^ and *n* = 6 for EC^ΔPGC1α^). Each dot represents one mouse. **g**, Expression of *MuRF1* mRNA levels in TA muscles (*n* = 12 for EC^WT^ and *n* = 7 for EC^ΔPGC1α^). Each dot represents one mouse. **h**, Representative 3D images of tdTomato^+^ (pseudocolored yellow) vessels in the GC muscles. **i**, Quantification of GC muscle vascular density (*n* = 6 for EC^WT^ and *n* = 9 for EC^ΔPGC1α^). Each dot represents one mouse. **j**, Representative images of muscle vascular leakage. **k**, Quantification of FITC–albumin for muscle vascular leakage in **j** (*n* = 5 for EC^WT^ and *n* = 3 for EC^ΔPGC1α^). Each dot represents one mouse. Data are presented as the mean ± s.e.m. Statistical analysis was conducted using an unpaired two-tailed *t*-test (**b**–**g**, **i** and **k**).

To examine whether downregulation of PGC1α in the endothelium was sufficient to adversely impact muscle function even in the absence of a tumor, we generated endothelial-specific inducible PGC1α-deletion mice (ECΔ^PGC1α^) (Fig. 7a,b). Notably, ECΔ^PGC1α^ mice phenocopied elements of cancer-induced cachexia symptoms such as reduced body weight, grip strength, muscle mass and CSA but increased expression of cachexia marker *MuRF1* (Fig. 7c–g). The ECΔ^PGC1α^ mice also presented significantly decreased muscle vascular density (Fig. 7h–i) and increased muscle capillary leakage (Fig. 7j,k) when compared to control mice. In addition, melanoma-bearing ECΔ^PGC1α^ mice exhibited further decreases in grip strength and muscle mass without affecting body weight when compared to melanoma-bearing EC^WT^ mice (Extended Data Fig. 8l–n). Taken together, these results suggest that downregulation of PGC1α in the endothelium induced by activin A in cancer cachexia causes loss of muscle vasculature and impairs vascular barrier integrity.

### Restoring EC function prevents cachexia

**Extended Data Fig. 9 |:**
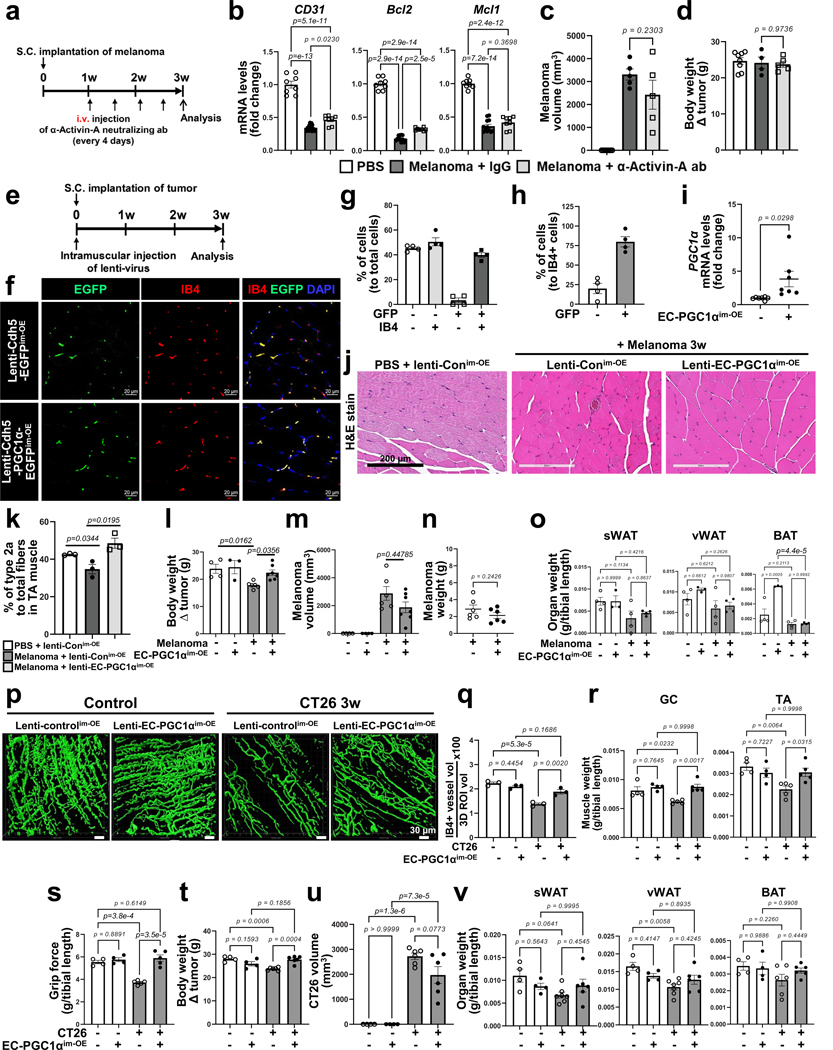
Preventive effect of local endothelial specific PGC1α overexpression in cancer cachexia progression. **a-d**. Effect of Activin-A neutralizing antibody. **a**. Timelines for Activin-A neutralizing antibody treatment. **b**. Expression of EC marker and anti-apoptotic genes in isolated muscle ECs (*CD31*; n = 8 for PBS, n = 12 for Melanoma + IgG, n = 8 for Melanoma + α-Activin-A ab, *Bcl2;* n = 8 for PBS, n = 12 for Melanoma + IgG, n = 8 for Melanoma + α-Activin-A ab, *Mcl1;* n = 8 for PBS, n = 12 for Melanoma + IgG, n = 8 for Melanoma + α-Activin-A ab). Each dot represents one mouse. **c**. The tumor growth (n = 4 for PBS, n = 5 for Melanoma + IgG and Melanoma + α-Activin-A ab). Each dot represents one mouse. **d**. The body weight was presented without (Δ) tumor weight (n = 8 for PBS, n = 4 for Melanoma + IgG, n = 5 for Melanoma + α-Activin-A ab). Each dot represents one mouse. **e**. Timelines for intramuscular injection of lenti-EC-PGC1α-GFP virus. **f-i**. Efficiency of local vascular overexpression of EC-PGC1α in muscles. **f**. All mice were perfused with IB4-A594 before harvesting the muscles, and the endothelial-specific expression of lenti-PGC1α-EGFP virus in muscle was determined by IF analysis for IB4. **g**. Percentage of GFP+ or IB4+ cells per field in **f** (n = 4). Each dot represents one mouse. **h**. Percentage of GFP+ cells in IB4+ cells in **f** (n = 4). Each dot represents one mouse. **i**. Expression of *PGC1α* in isolated muscle ECs (n = 7). **j-o**. Effect of local vascular overexpression of EC-PGC1α in melanoma bearing mice. **j**. H&E-stained GC muscles (n = 3). The ‘n’ represents the number of mice. **k**. Quantification of muscle fiber type 2a Fiber (n = 3). Each dot represents one mouse. **l**. Body weight was presented without (Δ) tumor weight (n = 4 for control, n = 3 for EC-PGC1α^im-OE^ alone, n = 6 for melanoma alone, n = 7 for melanoma with EC-PGC1α^im-OE^). Each dot represents one mouse. **m**. Melanoma growth (n = 4 for control, n = 3 for EC-PGC1α^im-OE^ alone, n = 6 for melanoma alone, n = 6 for melanoma with EC-PGC1α^im-OE^). Each dot represents one mouse. **n**. Melanoma weight (n = 6). Each dot represents one mouse. **o**. Adipose tissue weight (n = 4 for control, n = 3 for EC-PGC1α^im-OE^ alone, n = 4 for melanoma alone, n = 4 for melanoma with EC-PGC1α^im-OE^). Each dot represents one mouse. **p-v**. Effect of local overexpression of EC-PGC1α in CT26 bearing mice. **p**. 3D tissue images with IB4+ (green) functional vessels. **q**. Quantification of functional muscle vascular density (n = 3). Each dot represents one mouse. **r**. Muscle weight (n = 4 for control, n = 4 for EC-PGC1α^im-OE^ alone, n = 5 for CT26 alone, n = 5 for CT26 with EC-PGC1α^im-OE^). Each dot represents one mouse. **s**. Grip strength (n = 4 for control, n = 4 for EC-PGC1α^im-OE^ alone, n = 5 for CT26 alone, n = 5 for CT26 with EC-PGC1α^im-OE^). Each dot represents one mouse. **t**. Body weight without (Δ) tumor weight (n = 4 for control, n = 4 for EC-PGC1α^im-OE^ alone, n = 6 for CT26 alone, n = 6 for CT26 with EC-PGC1α^im-OE^). Each dot represents one mouse. **u**. CT26 tumor weight (n = 4 for control, n = 4 for EC-PGC1α^im-OE^ alone, n = 6 for CT26 alone, n = 6 for CT26 with EC-PGC1α^im-OE^). Each dot represents one mouse. **v**. Adipose tissue weight (n = 4 for control, n = 4 for EC-PGC1α^im-OE^ alone, n = 7 for CT26 alone for sWAT and vWAT, n = 6 for CT26 alone for BAT, n = 6 for CT26 with EC-PGC1α^im-OE^). Each dot represents one mouse. Data are presented as mean ± SEM. Statistical analysis was conducted using unpaired, two-tailed t-test (**i, n**) or one-way ANOVA with Tukey’s multiple comparison test (**b-d, k, l, m**, **o, q-v**).

**Fig. 8 |:**
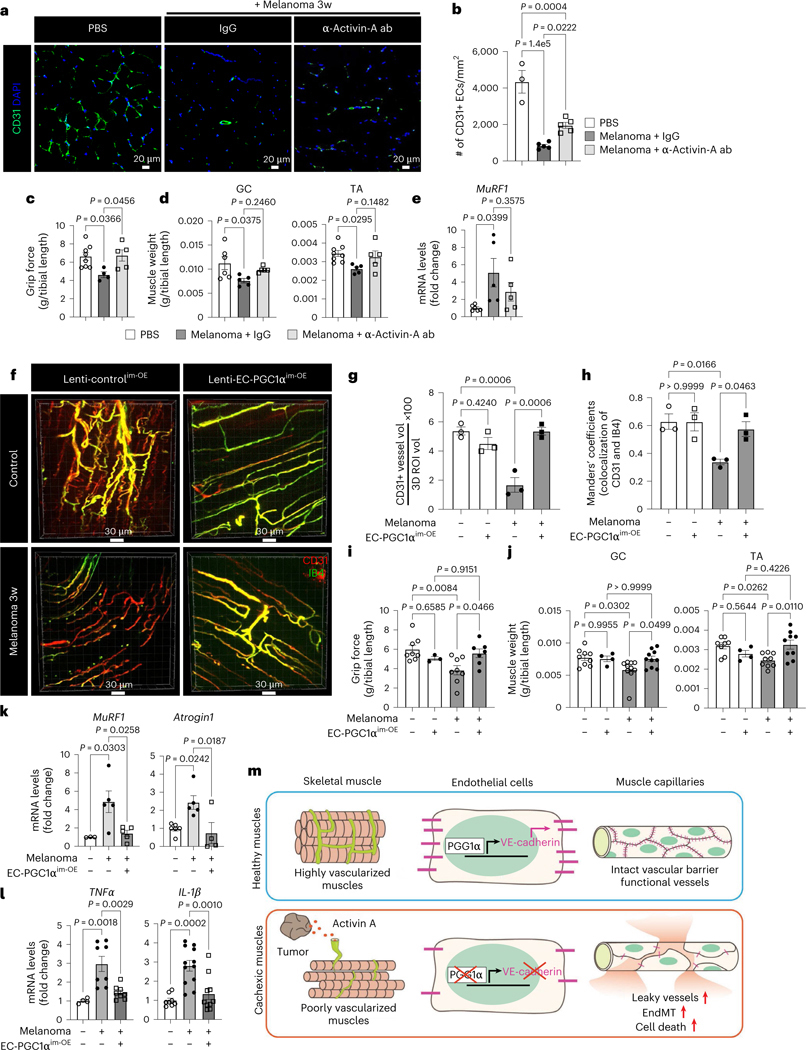
Targeting activin A–EC PGC1α axis prevents cancer cachexia progression. **a**–**e**, Mice were intravenously injected with anti-activin A neutralizing antibody or IgG1 isotype every 4 days, 1 week after melanoma implantation. **a**, Muscle vasculature by IF staining with CD31 antibody. Nuclei were stained with DAPI. **b**. Muscle vasculature density (*n* = 3 for PBS and *n* = 5 for melanoma + IgG and melanoma + anti-activin A antibody). Each dot represents one mouse. **c**, Mouse grip strength (*n* = 8 for PBS, *n* = 4 for melanoma + IgG and *n* = 5 for melanoma + anti-activin A antibody). Each dot represents one mouse. **d**, Muscle mass for GC (*n* = 6 for PBS and *n* = 5 for melanoma + IgG and melanoma + anti-activin A antibody) and TA (*n* = 8 for PBS and *n* = 5 for melanoma + IgG and melanoma + anti-activin A antibody). Each dot represents one mouse. **e**, Cachectic marker *MuRF1* mRNA in TA muscle (*n* = 6 for PBS and *n* = 5 for melanoma + IgG and melanoma + anti-activin A antibody). Each dot represents one mouse. **f**–**l**, After melanoma implantation, the mice were intramuscularly injected with lentiviral control and lenti-EC PGC1α–GFP. **f**–**h**, Mice were retro-orbitally injected with IB4–A594 before isolating muscles. **f**, Representative 3D vasculature after overlaying with CD31^+^ (red) and IB4^+^ (pseudocolored green) vessels in GC muscles. **g**, Quantification of CD31^+^ (red) muscle vasculature density in **f** (*n* = 3). Each dot represents one mouse. **h**, Manders’ colocalization coefficients for CD31 and IB4 in **f** (*n* = 3). Each dot represents one mouse. **i**, Mouse grip strength (*n* = 8 for control, *n* = 3 for EC PGC1α^im-OE^ alone, *n* = 8 for melanoma alone and *n* = 7 for melanoma with EC PGC1α^im-OE^). Each dot represents one mouse. **j**, Muscle mass for GC (*n* = 9 for control, *n* = 4 for EC PGC1α^im-OE^ alone, *n* = 10 for melanoma alone and *n* = 10 for melanoma with EC PGC1α^im-OE^) and TA (*n* = 8 for control, *n* = 4 for EC PGC1α^im-OE^ alone, *n* = 9 for melanoma alone and *n* = 9 for melanoma with EC PGC1α^im-OE^). Each dot represents one mouse. **k**, mRNA levels of *MuRF1* (*n* = 3 for control and *n* = 5 for melanoma with or without EC PGC1α^im-OE^) and *Atrogin1* (*n* = 6 for control, *n* = 5 for melanoma without EC PGC1α^im-OE^ and *n* = 4 for melanoma with EC PGC1α^im-OE^) by RT–qPCR. Each dot represents one mouse. **l**, Inflammatory genes in GC muscles by RT–qPCR (*TNF*, *n* = 4 for control and *n* = 8 for melanoma with or without EC PGC1α^im-OE^; *IL1β*, *n* = 8 for control, *n* = 12 for melanoma without EC PGC1α^im-OE^ and *n* = 10 for melanoma with EC PGC1α^im-OE^). Each dot represents one mouse. **m**, Graphical summary. All mice were examined 3 weeks after melanoma implantation. Data are presented as the mean ± s.e.m. Each gene level was normalized by *PPIA* levels and is presented as the FC. Statistical analysis was conducted using a one-way ANOVA with Tukey’s multiple-comparison tests (**b**–**e** and **g**–**l**).

To assess the therapeutic effects of inhibiting the activin A–PGC1α axis in the muscle endothelium, we first examined whether inhibition of activin A signaling using an anti-activin A neutralizing antibody could restore EC function and prevent muscle loss in cancer cachexia (Extended Data Fig. 9a). Activin A neutralization through systemic intravenous injection of the antibody significantly increased the number of ECs and the mRNA levels of the EC marker *CD31* and antiapoptotic *Bcl2* genes compared to injection of control IgG isotype antibodies in melanoma-bearing mice (Fig. 8a,b and Extended Data Fig. 9b). Activin A neutralization ultimately rescued mouse grip strength, increased muscle mass and showed a trend toward decreased expression of the cachexic marker *MuRF1* (Fig. 8c–e). However, the anti-activin A neutralizing antibody alone did not completely restore vascular density (Fig. 8b) or affect tumor growth and body weight (Extended Data Fig. 9c,d).

Next, we examined whether restoring EC function by local or systemic overexpression of EC PGC1α could prevent cancer cachexia. We generated a lentiviral construct (lenti-*Cdh5*–mPGC1α–*cmv*–GFP virus, hereafter referred to as lenti-EC PGC1α–GFP) to overexpress PGC1α specifically in ECs. After melanoma implantation, the mice were intramuscularly injected with lenti-EC PGC1α–GFP, which was specifically expressed in the muscle endothelium as confirmed by the colocalization of IB4 and GFP (Extended Data Fig. 9e–h) and increase in *PGC1α* mRNA levels (Extended Data Fig. 9i). EC PGC1α intramuscular overexpression (^im-OE^) preserved the total CD31^+^ muscle vascular density (Fig. 8f–g), which was reduced in control^im-OE^ melanoma-bearing mice. Importantly, EC PGC1α overexpression in melanoma-bearing mice increased functional vessels, as evaluated by the Manders’ coefficients for colocalization of the endothelial marker CD31 and the perfusion marker IB4 (Fig. 8h). Mouse grip strength and muscle mass were also increased by EC PGC1α^im-OE^ in melanoma-bearing mice (Fig. 8i–j). Moreover, EC PGC1α^im-OE^ preserved muscle CSA (Extended Data Fig. 9j), decreased expression of cachexia marker genes, *MuRF1* and *Atrogin1* (Fig. 8k), and restored the number of type 2a muscle fibers and body weight (Extended Data Fig. 9k–l). Additionally, EC PGC1α^im-OE^ also prevented the upregulation of inflammatory genes in the TA muscle of melanoma-bearing mice (Fig. 8l) without altering tumor growth, tumor weight and adipose tissues (Extended Data Fig. 9m–o). We next assessed whether the benefits of EC PGC1α overexpression were also seen in other cancer cachexia models. EC PGC1α^im-OE^ in CT26-bearing mice also significantly prevented the loss of functional IB4^+^ muscular vessels, mouse grip strength, muscle mass and body weight (Extended Data Fig. 9p–t) without altering tumor growth and adipose mass when compared to control^im-OE^ CT26-bearing mice (Extended Data Fig. 9u–v).

**Extended Data Fig. 10 |:**
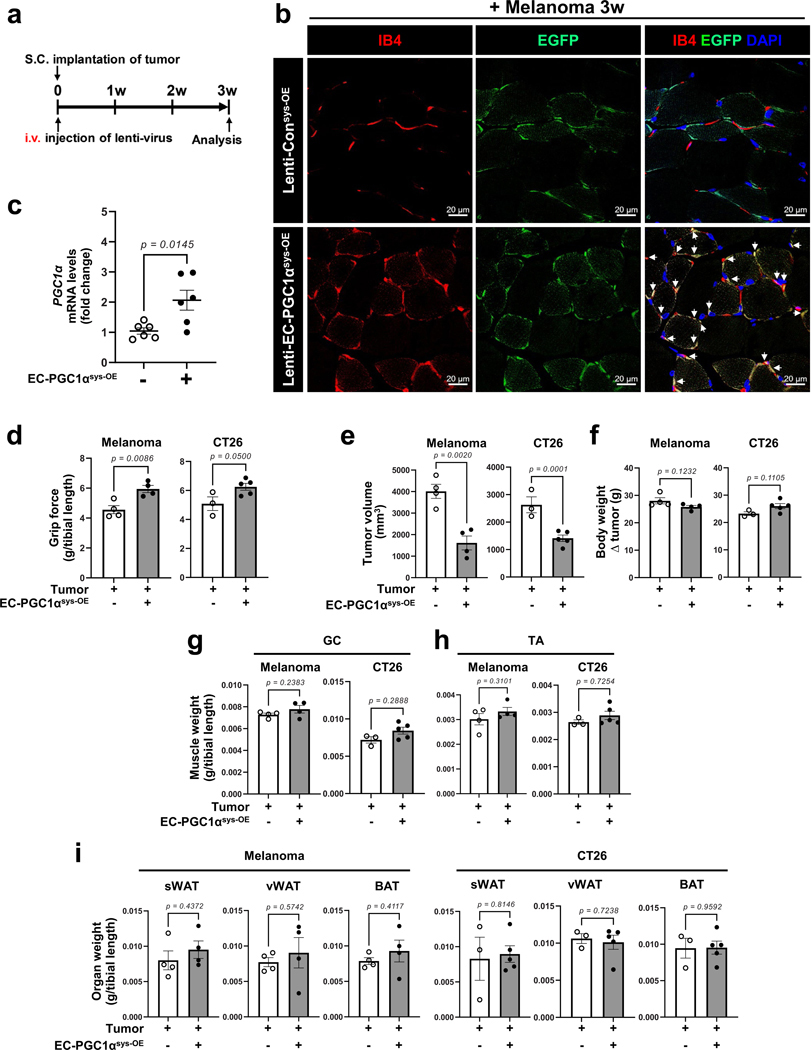
Effect of systemic endothelial specific PGC1α overexpression in cancer cachexia progression. **a**. Timelines for systemic injection of EC-PGC1α lentivirus. The ‘^sys-OE’^ indicates the systematical overexpression. **b-i**. After tumor implantation, the mice were intravenously injected with lenti-control and lenti-PGC1α-EGFP virus and evaluated 3 weeks later. **b**. IB4 positive (+) functional vessels in gastrocnemius muscle of control and melanoma 3w mice (n = 4). **c**. Expression levels of *PGC1α* in isolated muscle ECs (n = 6). Each dot represents one mouse. **d-i**. melanoma-bearing mice (n = 4) and CT26-bearing mice (n = 3 for CT26 alone, n = 5 for CT26 with EC-PGC1α^sys-OE^). **d**. Mouse grip strength. **e**. Tumor growth. **f**. Body weight without (Δ) tumor weight. **g**. GC muscle weight. **h**. TA muscle weight. **i**. Adipose tissues’ weight. Data are presented as mean ± SEM. Statistical analysis was conducted using an unpaired, two-tailed t-test (**c-i**).

Next, we examined whether restoring EC function by systemic overexpression of EC PGC1α could prevent cancer cachexia. After tumor implantation, the mice were intravenously injected with lenti-EC PGC1α–GFP (Extended Data Fig. 10a). The EC PGC1α systemic overexpression (EC PGC1α^sys-OE^) also preserved IB4^+^ functional muscle vessels (Extended Data Fig. 10b) by increasing expression of PGC1α in muscle endothelium (Extended Data Fig. 10c) and restored mouse grip strength (Extended Data Fig. 10d) in B16F10-cachexia and CT26-cachexia mouse models. Interestingly, the EC PGC1α^sys-OE^ tumor-bearing mice had significantly inhibited tumor growth compared to control^sys-OE^ tumor-bearing mice (Extended Data Fig. 10e). However, the systemic overexpression of EC PGC1α did not affect body weight and mass of muscle or adipose tissues (Extended Data Fig. 10f–i). These results suggest that improvement of muscle vascular health by overexpressing EC PGC1α intramuscularly can delay the loss of muscle mass and function but has only a modest beneficial effect on tumor growth. Systemic therapy by overexpressing EC PGC1α inhibits tumor growth but has a less pronounced therapeutic benefit for cachexia progression.

Taken together, we propose that tumor-released circulating activin A remotely suppresses the expression of PGC1α in muscle endothelium and results in endothelial dysfunction by increasing EC death, vascular leakiness and EndMT, thereby causing cachexic muscles (Fig. 8m).

## Discussion

Here we identified muscle endothelial dysfunction as a hallmark of cancer cachexia and a precursor to muscle atrophy. Tumor-derived activin A is released into the circulation, where it acts remotely on the muscle endothelium to induce barrier disruption, apoptosis and EndMT. Impaired muscle vascular function then results in muscle atrophy and cachexia. Similar to the emerging concept of a ‘premetastatic niche’, which purports that tumors can prime distant vascular beds for future metastases, we propose that tumors also remotely act on the muscle vasculature to create a ‘precachectic niche’ before the development of cachexia.

A previous study found that TNF contributes to cancer cachexia by stimulating Notch 1 signaling in white adipose endothelium^49^. These findings suggest heterogeneity in the response to tumor-derived factors amongst the vascular beds, with the vascular endothelium of muscle and adipose tissues potentially being more susceptible to cachexia mediators. Cachexia-mediated endothelial loss may also depend on disease stage and severity, as an earlier study noted no significant change in muscle capillary density^50^. We used a tissue-clearing method for 3D high-resolution microscopy that enabled comprehensive visualization of the muscle vascular network dynamics during disease progression. We found that loss of muscle vascular density in cancer cachexia mouse models was primarily accompanied by a reduction of type 2a fibers. Several studies have shown that cancer cachexia can affect both type 1 and type 2 fibers dependent on the severity of the disease and location of the tumor^17,51–53^. It is therefore possible that the contribution of vascular dysfunction to the loss of specific muscle fibers may also vary on the basis of the type and location of tumors.

We observed that the underlying mechanisms of low muscle vascular density in cancer cachexia include increased endothelial death and EndMT during cachexia progression. Mechanistically, muscle vascular dysfunction can cause muscle loss because of impaired perfusion, increased hypoxia, increased expression of inflammatory cytokine genes and enhanced immune cell infiltration through a compromised vascular barrier. Our findings of vascular leakiness are consistent with other reports on a compromised blood–brain barrier^54,55^ and higher vascular leakiness in TA muscles in CT26 tumor-bearing mice when compared to control mice^56^. Taken together, our results suggest that the loss of functional muscle vasculature through apoptosis and EndMT during the early phases of tumor progression creates a local milieu of inflammation and impaired perfusion, which, in turn, could promote muscle loss.

We observed the emergence of a relatively small muscle EC subpopulation (Cap2) in B16F10-cachexia mice using single-cell transcriptomic analysis. Activin A target genes were expressed at higher levels in multiple additional muscle EC populations of tumor-bearing mice and broadly correlated with higher muscle differentiation scores, consistent with the notion that activin A induces EndMT, indicating that activin A-induced dysfunction is not limited to the smaller Cap2 population. We also found that activin A overexpression in non-tumor-bearing mice induced skeletal muscle endothelial dysfunction. Increased expression of activin receptors in the endothelium when compared to the other cell types in muscles may explain why the muscle endothelium is more susceptible to circulating activin A. The downstream effects of activin A may vary dependent on the specific receptors expressed in various tissues and cell types. Moreover, additional factors released by tumors may further exacerbate the extent of cachexia and lead to more devastating phenotypes^57–60^.

There has recently been a shift away from traditional antiangiogenic therapies toward tumor vessel normalization approaches^61^. Interestingly, vessel normalization improves vessel function^62,63^, increases anticancer drug delivery^8^, promotes immunotherapy efficacy^64^ and complements traditional antiangiogenic approaches. Our findings suggest the value of a parallel approach that induces muscle vessel normalization and, thus, prevents cachexia in the presence of tumors. We found that therapeutic administration of anti-activin A neutralizing antibody significantly rescued mouse grip strength, complementing the results of a previous report that soluble activin type IIB receptor reduced cachexia in mice with high-activin tumors^36^. However, the antibody alone was insufficient for the complete restoration of vascular density, suggesting that broader downstream changes may occur in ECs soon after tumor formation, which could not be rescued by the antibody. Importantly, ECΔ^PGC1α^ mice manifest some aspects of cancer cachexia even in the absence of tumors by showing loss of muscle vascular density and impaired muscle function. In contrast, overexpression of PGC1α specifically in the muscle endothelium could rescue grip strength and muscle mass in tumor-bearing mice and showed a trend toward decreased tumor growth, but we observed significant variability among individual mice. We also found that systemic overexpression of EC PGC1α in whole-body endothelium restored grip strength in tumor-bearing mice and reduced tumor growth. However, the underlying mechanisms by which targeting the activin A–PGC1α axis reduces tumor progression will need to be investigated in future studies. Endothelial-specific delivery of PGC1α using nanoparticles was also reported to prevent hypoxia-induced pulmonary hypertension through inhibition of EndMT-induced vascular remodeling^65^, thus mirroring what we observed in the cancer cachexia setting. It was previously shown that increased expression of PGC1α in the muscle tissue also enhanced muscle function in elderly mice^66^ and maintained muscle mass in a cancer cachexia mouse model^67^. The roles of PGC1α in the vascular endothelium and muscle may also vary depending on the specific tissue and disease context^68,69^. These findings highlight the need for specifically targeting distinct cell populations in a complex disease such as cachexia to prevent disease progression.

We observed a loss of muscle endothelium in autopsy muscle sections of participants with cancer, suggesting that our findings in the experimental tumor models have relevance for human disease. However, it is important to note that clinical muscle samples from autopsies show substantial heterogeneity and that additional studies in humans are required to more precisely define the onset and extent of muscle endothelial dysfunction during cachexia progression. Another limitation of our study is that we relied on body mass index (BMI) to define cachexia. Furthermore, not all malignancies induce cachexia and future studies could use cachexia-inducing cancer cell lines and non-cachexia-inducing cancer cell lines to establish molecular signatures that are differentially correlated with muscle vascular function and density during tumor growth.

In summary, our findings establish the importance of skeletal muscle endothelial dysfunction as a key early pathogenic mediator in cancer cachexia, which could serve as an important therapeutic target to prevent or reverse cachexia in persons with cancer.

## Methods

### Ethics

All animal experiments were performed in accordance with institutional and national regulations and were approved by the Animal Care and Institutional Biosafety Committee of the University of Illinois Chicago (UIC; ACC 24–084). Analysis of human samples was deemed to not constitute research on human subjects by the UIC Office for the Protection of Research Subjects because the obtained muscle sections were provided as deidentified formalin-fixed paraffin-embedded blocks from the UIC Pathology Biorepository. We did not collect any data through interactions or interventions with individuals for research purposes and we did not use any private, identifiable information about subjects.

### Mice

Mice were maintained under standard conditions (standard diet and water) at 23 °C and ~60% humidity with 12-h light–dark cycles. We purchased C57BL/6J (000664) or BALB/cJ (000651) mice from Jackson Laboratory as control mice. KPC mice were generated by crossing *LSL-Kras*^G12D/+^:*LSL-Trp53*^R172H/R172H^ mice with *Pdx1-Cre* homozygous mice^13^. We used inducible endothelial-specific tdTomato-expressing mice (tdTomato flfl;Cdh5-Cre^ERT2^,EC^tdTomato^)^70^ for scRNA-seq analysis. We generated the endothelial-specific inducible PGC1α knockout mice (PGC1α flfl;Cdh5-Cre^ERT2^ and PGC1α flfl;tdTomato flfl;Cdh5-Cre^ERT2^) by crossing PGC1α flfl mice (009666, Jackson Laboratory) with Cdh5-Cre^ERT2^ mice or tdTomato flfl;Cdh5-Cre^ERT2^ mice. We used both male and female mice in our experiments.

#### Tumor syngeneic allograft assay.

Skin melanoma B16F10 cells (American Type Culture Collection (ATCC), CRL-6475; 10^6^ cells per 100 μl of PBS) or Lewis lung carcinoma LLC1 cells (ATCC, CRL-1642; 10^6^ cells per 100 μl of PBS) were implanted subcutaneously in the dorsal flank of 10-week-old C57BL/6J male and female mice. CT26 colon carcinoma cells (ATCC, CRL-2639; 10^6^ cells per 100 μl of PBS) were implanted subcutaneously in the dorsal flank of 10-week-old BALB/cJ WT male mice. Control mice were injected with PBS. The tumor size was assessed by tumor volume, which was calculated as width^2^ × height × 0.523, as previously described^71^. The maximum allowable tumor diameter was limited to 2 cm, in accordance with the guidelines established by our institutional review board. The tumor size did not exceed 2 cm in diameter in this study.

#### Effect of anti-activin A neutralizing antibody in cancer cachexia.

Mice aged 10–14 weeks were treated systemically with anti-activin A neutralizing antibody (R&D systems, MAB3381; 10 mg kg^−1^ body weight) 1 week after tumor implantation through the tail vein every 4 days. The same dose of IgG1 isotype control (R&D systems, MAB002) was used as the control antibody. All mice were examined at 3 weeks after tumor implantation.

#### Effect of overexpression of EC-specific PGC1α in vasculature.

We generated lentivirus expressing mouse PGC1α under endothelial-specific Cdh5 (*CD144*) promoter. To assess the local effect of EC PGC1α, the mice aged 10–14 weeks were injected intramuscularly with a total of 200 μl of lentivirus (1 × 10^9^ plaque-forming units per μl, 40 μl per injection site) at five different areas into the TA and GC of hindlimbs right after tumor implantation. To assess the systemic effect of EC PGC1α, the mice were injected retro-orbitally with 100 μl of lentivirus (1.2 × 10^7^ transducing infectious units (TIU)) right after tumor implantation. Lentivirus-expressing empty vectors or EGFP-expressing vectors were used as controls. All mice were examined at 3 weeks after tumor implantation.

#### Effect of overexpression of AAV-activin A in muscle vasculature.

We generated an AAV expressing mouse activin A. To assess the systemic impact of AAV-activin A, the mice aged 10–14 weeks were injected retro-orbitally with 100 μl of AAV in a dose-dependent manner (0, 1 × 10^12^, 1 × 10^13^ and 1 × 10^14^ TIU). The expression of activin A was determined in blood plasma once per week for 3 weeks using an activin A ELISA kit (DAC00B).

#### Mouse body composition.

Control and tumor-bearing mice were measured body weight before nuclear magnetic resonance (NMR) analysis. The mouse body composition was measured using a Bruker NMR mini-spec just before euthanasia. The lean, fat or free body fluid mass was presented as absolute values per mouse without normalization.

#### Mouse grip strength.

Grip strength in all four limbs (forelimbs and hindlimbs) was measured using a Bio-GS3 grip strength test meter^72^ with a wire mesh grid for four paws^36,73–75^. Each mouse, aged 10–14 weeks, was measured five times consecutively with a 30-s pause between each measurement. The values were averaged to reduce procedure-related variability^76^ and the data were presented as averages after normalization against tibial length, which is not altered by muscle loss^77,78^.

#### Mouse tissue collection.

We measured mouse body weight and tibial length before dissecting tissues with a digital caliper. Skeletal muscles (GC, TA or quadriceps) from both hindlimbs and adipose tissues (subcutaneous white adipose tissue, visceral white adipose tissue or brown adipose tissue) were dissected and immediately weighed using a microelectronic weighing scale (Accuris instruments, W3100A-120) and then directly embedded in tissue plus optimal cutting temperature (OCT) compound (Fisher) on dry ice for cryosectioning. For paraffin blocks, the tissues were fixed with 10% neutral buffered formalin overnight at 4 °C and changed with 70% ethanol following paraffin embedding. The tissue mass (weight, g) was normalized using tibial length (mm). For western blotting or reverse transcription (RT)–qPCR, the muscles were immediately frozen in liquid nitrogen and stored at −80 °C until use.

### Mouse skeletal muscle EC isolation

Skeletal muscles (GC, TA or quadriceps) from hindlimbs were dissected, minced and digested with digestion buffer (2 mg ml^−1^ collagenase A, 1 mg ml^−1^ dispase II and 0.1 mg ml^−1^ DNase I in 1× PBS without Ca^2+^ or Mg^2+^) at 37 °C for 30 min with gentle shaking. To evaluate a relative number of muscle ECs in total muscle cells, the same number of cells (10^6^ cells per 100 μl) were stained with specific antibodies to CD31 (EC marker, eBiosciences 17–0311-82; 1:100) and CD45 (immune cell marker, BioLegend 103108; 1:100) for 30 min on ice. Samples were run through a Gallios flow cytometer (Beckman Coulter) and analyzed by Kaluza software (Beckman Coulter). The muscle ECs (CD31^+^CD45^−^DAPI^−^) from a single-cell suspension of total muscle cells were obtained by fluorescence-activated cell sorting (FACS) using MoFlo Astrios (Beckman Coulter) or isolated using mouse CD31 microbeads (130–097-418, Miltenyi Biotec) and an MS column (130–042-201, Miltenyi Biotec) with a magnetic separator for some experiments.

### Muscle clearing and 3D imaging

GC muscles were freshly isolated and fixed with 2% PFA for 10 min at room temperature. After washing in PBS, the muscles were longitudinally sectioned to 400-μm thickness using a vibratome (Leica, VT1200S) and stained with primary rat anti-mouse CD31 antibody (BioLegend, 102502; 1:1,000 dilution) for 18 h at 4 °C. Then, the tissues were incubated with fluorescent secondary anti-rat IgG2 antibody at a 1:100 dilution for 18 h at 4 °C. The secondary antibody was prepared by conjugating anti-rat IgG2 antibody (BioLegend, 407502) with DyLight 633 (Thermo Fisher, 46414) at a 1:10 ratio for 18 h at 4 °C. For optical tissue clearing, the muscle tissues were incubated in a gradient d-fructose solution (20% for 30 min, 80% for 30 min and 100% for 1 h) at room temperature^15^. The 3D images were taken using a confocal fluorescence microscope (Caliber ID, RS-G4) with a ×40 oil objective (Olympus UPLXAPO 40XO, 1.4 numerical aperture (NA), 0.13 mm working distance). The light source was 640-nm excitation (Toptica iChrome MLE-LFA 50-mW diode laser) with a 630/69-nm filter (Semrock) for DyLight 633. The high-resolution 3D images were obtained from 6–9 different muscle tissue areas and reconstructed for visualization with Imaris Viewer 64-bit version 9.6.0. The surface volume of vessels was analyzed with Imaris 64-bit version 7.2.2. Muscle vascular density is presented as a percentage of CD31^+^ vessel surface volume in the 3D region of interest (ROI).

### IF imaging using confocal microscopy

#### Muscle tissue imaging for cryosection.

The 7-μm cryosections of muscle were dried for 5 min at room temperature and washed with 1× PBS for 5 min. The sections were blocked with blocking buffer (1× PBS, 2% BSA, 0.05% Tween-20 and 5% goat serum) for 1 h at room temperature and then stained with EC-specific anti-CD31 antibody (BD biosciences, 550274; 1:25 dilution) or muscle basal membrane marker anti-laminin antibody (Sigma, L9393; 1:100 dilution) overnight at 4 °C in a humidified chamber. The secondary antibodies used were goat anti-rat IgM cross-adsorbed secondary antibody DyLight 488 (Thermo Fisher, SA5–10010, 1:400 dilution) or goat anti-rabbit IgG (H + L) cross-adsorbed secondary antibody Alexa Fluor 633 (Thermo Fisher, A21070; 1:500 dilution) for 1 h at room temperature. The nuclei were stained with DAPI. The images were taken by confocal microscopy (LSM710 Meta or LSM900, ×20 objective).

#### Muscle tissue imaging for paraffin section.

The 4-μm cross-paraffin sections of muscle were deparaffinized and hydrated at room temperature as follows: 100% xylene 1, 5 min; 100% xylene 2, 5 min; 100% ethanol, 3 min; 95% ethanol, 3 min; 70% ethanol, 3 min; 50% ethanol, 3 min; distilled water, 5 min; Tris-buffered saline with 0.1% Tween-20, 5 min. For antigen retrieval, the slides were boiled in sodium citrate retrieval buffer (pH 6.0, 0.05% Tween-20) for 30 min and cooled for 20 min at room temperature. After washing with 1× PBS, the slides were permeabilized with 0.2% Triton X-100 for 10 min at room temperature and followed by blocking with blocking buffer (1× PBS, 2% BSA, 0.05% Tween-20 and 5% goat serum) for 1 h at room temperature and then stained with EC-specific CD31 antibody (ab28364; 1:100 dilution) or biotinylated IB4 (Vector Labs, B-1205-.5; 1:100 dilution) overnight at 4 °C, followed by staining with goat anti-rabbit Alexa Fluor 594 secondary antibody (A-11012, Invitrogen; 1:500 dilution) or FITC–streptavidin (Invitrogen, 11–4317-87; 1:500 dilution) for 1 h at room temperature. The images were taken by confocal microscopy (LSM880 or LSM710 Meta, ×20 or ×40 objectives).

#### Muscle fiber type determination.

Fiber type staining was performed as described previously^34,79^ using the 5-μm cross-cryosections of GC or TA muscles with myofiber type-specific primary antibodies: type 1 for slow oxidative fiber with BA-D5 (antibody isotype MIgG2b, 1:50 dilution), type 2a for fast oxidative fiber with sc-71 (antibody isotype MIgG1, 1:100 dilution) and type 2b for fast glycolytic fiber with BF-F3 (antibody isotype MIgM, 1:50 dilution) antibody from DSHB for overnight at 4 °C, followed by staining with goat anti-mouse IgG2b Alexa Fluor546 secondary antibody (A-21143, 1:400 dilution), rat anti-mouse IgG1–FITC secondary antibody (11–4015-82, 1:400 dilution), goat anti-mouse IgM (heavy chain) Alexa Fluor 633 secondary antibody (A21046, 1:400 dilution) or goat anti-mouse IgM DyLight650 secondary antibody (SA5–10153, 1:400 dilution) for 1 h at room temperature. The images were taken by confocal microscopy (LSM900, ×20 objectives).

#### In vitro cell imaging.

The primary HLMVECs were cultured on coverslips in six-well plates. After treatment with lentiviral short hairpin RNA (shRNA) for indicated times, the cells were fixed with 4% PFA for 10 min at room temperature and then blocked with blocking buffer (1× PBS, 2% BSA and 0.05% Tween-20) without permeabilization for 1 h. EC barrier integrity was visualized by immunostaining with 1:250 dilution of Alexa Fluor647 mouse anti-human CD144 (BD561567) for 1 h at room temperature. After mounting with antifade reagent with DAPI (Vector Labs, H-1800–10), the images were taken using confocal microscopy (Zeiss LSM880, Plan Apo 1.46 NA, ×63 objective).

### Human biospecimens and pathology evaluation

Biospecimens were collected as deidentified specimens. We included both male and female specimens, as determined by assignment. Archival paraffin blocks of skeletal muscles were obtained from the University of Illinois Health System. Rectus abdominis blocks were obtained from autopsy cases in cases documented with cancer or no cancer and participants with cancer were further stratified by clinical definition of cachexia as previously described^27^. Specifically, diagnostic criteria for cachexia in participants with cancer were as follows: (1) weight loss > 5% over a 6-month period or (2) BMI < 20 with >2% weight loss in a 6-month period. For the calculation of the weight loss percentage, we used the percentage change of BMI as BMI is calculated as body weight in kg/(height in m)^2^ and body height is expected to be stable. In Supplementary Tables 1–7, the cachexia diagnosis section shows information for each case, listing an initial BMI (BMI1) = BMI (kg/m^2^) determined during the 6-month period before death and the subsequent BMI (BMI2) = BMI (kg/m^2^) determined during the perimortem period. All histology specimens were reviewed by two independent pathologists. Basic demographic information of participants is available in Supplementary Table 7. We did not analyze between-sex differences in this study.

#### Immunohistochemistry and IF assay in human muscle sections Immunohistochemistry.

The paraffin sections (4 μm) were stained with hematoxylin and eosin (H&E) and Masson trichrome by the UIC histology core laboratory. The whole-tissue images were scanned using an Aperio AT2 microscope from Leica Biosystems with bright-field ×20 or ×40 objectives and analyzed by ImageScopex64 software. The quantification of atrophy and fibrosis in skeletal muscle sections was also qualitatively confirmed by two independent pathologists in a blinded manner. For the IF assay, ECs in muscles were stained with monoclonal mouse anti-human CD31 antibody (EC clone JC70A, M0823, Dako; 1:100 dilution) or with polyclonal rabbit anti-VE-cadherin (Cayman, 160840; 1:100 dilution) overnight at 4 °C. The sections were followed for staining with goat anti-mouse Alexa Fluor 488 secondary antibody (A-21121, Invitrogen; 1:500 dilution) or with goat anti-rabbit Alexa Fluor 647 secondary antibody (A-21245, Invitrogen; 1:500 dilution). Nuclei were stained with DAPI. The images were taken using confocal microscopy (LSM710 Meta; ×40 objective, LSM900; ×20 objective).

### Muscle capillary permeability assay

WT and cancer cachexia mice were anesthetized with ketamine and xylazine and retro-orbitally administered FITC–albumin (250 mg kg^−1^ in PBS)^80^ or fixable FITC–dextran (Biotium, 80126, 0.5 mg per 100 μl of PBS). After 10 min, circulating FITC–albumin was washed away by PBS perfusion and skeletal muscles (GC and TA) were immediately dissected and directly embedded in tissue plus OCT compound on dry ice for cryosection. The frozen muscles were sectioned with 50-μm thickness using a cryostat and dried for 5 min at room temperature, followed by a PBS wash and direct mounting with antifade reagent with DAPI (Vector Labs, H-1800–10). The FITC–albumin and FITC–dextran fluorescence images were taken by *Z*-sectioning using confocal microscopy (LSM710 or LSM900, ×20 objective) and reconstructed for visualization using Imaris 64-bit 7.2.2. The surface volume of FITC–albumin fluorescence in muscle was measured using Imaris 64-bit 7.2.2 and the fluorescence intensity of FITC in muscle was measured using Image J.

### Functional vessel analysis

Control and cancer cachexia mice were anesthetized with ketamine and xylazine and were injected retro-orbitally with IB4, DyLight594 (IB4–A594, Vector Labs, DL-1207-.5; 50 μg per 100 μl in 1 mM CaCl_2_). After 30 min, circulating IB4 was washed away by PBS perfusion and skeletal muscles (GC and TA) were immediately dissected. For 3D tissue imaging, the muscles were processed for fixation and tissue clearing before imaging, as mentioned above. For 2D images, the muscles were directly embedded in tissue plus OCT compound on dry ice for cryosection. The frozen muscles were sectioned with 50-μm thickness using a cryostat and dried for 5 min at room temperature, followed by a PBS wash and direct mounting with antifade reagent with DAPI (Vector Labs, H-1800–10). The IB4–A594 fluorescence images were taken by *Z*-sectioning using confocal microscopy (LSM710, ×40 objective) and reconstructed for visualization using Imaris 64-bit 7.2.2.

### Muscle hypoxia assay

Mice were intraperitoneally injected with hypoxic probe pimonidazole (100 mg kg^−1^, Hypoxyprobe, Hypoxyprobe plus kit) 1 h before isolating muscle^81^. The 50-μm cryosections or 4-μm paraffin sections were used to measure the extent of hypoxia in the GC muscle by following the manufacturer’s instructions. Nuclei were stained with DAPI. The FITC–pimonidazole fluorescence images were taken by *Z*-sectioning using confocal microscopy (LSM710, ×20 objective) and reconstructed for visualization using Imaris 64-bit 7.2.2. The surface volume of FITC fluorescence in muscle was measured using Imaris 64-bit 7.2.2 and the fluorescence intensity of FITC in muscle was measured using Image J.

### In situ terminal deoxynucleotidyl transferase dUTP nick-end labeling assay

To determine apoptotic ECs in muscles from cancer cachexia mice, the paraffin sections were deparaffinized and hydrated and antigen retrieval was performed. The apoptotic cells in sections were determined using the terminal deoxynucleotidyl transferase dUTP nick-end labeling (TUNEL) assay kit for in situ apoptosis detection (Invitrogen, C10618) costained with EC-specific TF early growth response 1 (EGR1) antibody by following the manufacturers’ instructions with minor modifications.

### Flow cytometric analysis

#### For infiltrated immune cells in muscle.

Single-cell suspensions were counted and the same number of cells (10^6^ cells per 100 μl) were stained with a 1:100 dilution of anti-CD45 (157610, BioLegend) antibody and DAPI. Samples were run through a Gallios flow cytometer (Beckman Coulter) and analyzed by Kaluza software (Beckman Coulter). The inflammatory response (CD45^+^DAPI^−^) cells was presented as the percentage of total muscle cells. For cell apoptosis, cells were stained with annexin V–FITC and PI (Bio-Rad, ANNEX20F) following flow cytometric analysis using a Gallios flow cytometer (Beckman Coulter).

### Blood plasma activin A level assessment by ELISA

Mouse whole blood was slowly withdrawn by cardiac puncture and collected in tubes containing anticoagulant (0.5 M EDTA, 5 μl per 100 μl of blood). The blood was centrifuged at 2,000*g* at 4 °C for 15 min and plasma was collected in new tubes and stored at −80 °C until use. Activin A levels were measured with 100 μl of plasma for each sample using ELISA Kits (DAC00B).

### shRNA, plasmid and lentivirus production

Five lenti-shPGC1α RNA constructs were purchased from Sigma (TRCN0000364084, TRCN0000364085, TRCN0000364086, TRCN0000001166 and TRCN0000001167) and knockdown efficiency was examined in ECs. The TRCN0000001166 clone showed the best effect to deplete PGC1α in ECs and we used this clone for experiments. pcDNA4-myc-PGC1α (plasmid 10974) and PGC1α promoter luciferase delta CRE (plasmid 8888) were purchased from Addgene. pRL/TK (*Renilla* luciferase) was provided by C. Tiruppathi at UIC. Mouse endothelial-specific lentiviral vectors for PGC1α (pLV-EGFP-Cd144_mPpargc1α (NM_008904.2)) were designed and produced by Vector Builder. Lentiviruses were produced in HEK293T cells (ATCC, CRL11268) as described previously^82^ by transfecting with DNA (2.5 μg pMD2.G, 5 μg of psPAX2 and 7.5 μg of DNA expression vector) and 30 μg of polyethylenimine (PEI; Polysciences, 23966). To assess the systemic effect of EC PGC1α overexpression, the lentiviruses were purified by cesium chloride density-gradient ultracentrifugation and titrated as TIU by the viral core facility at UIC.

### Primary EC culture

HLMVECs (CC-2527, Lonza) were obtained from Lonza and cultured with EGM2 (Lonza) including all supplements and 10% FBS (Hyclone) until passage 8. The cells were transduced with lenti-shPGC1α RNA virus with 1:2,000 dilution polybrene (Millipore, TR-1003-G) for 24 h and then the medium was changed. Cells were used for experiments at 72 h following virus infection. For activin treatment, cells were starved with 2% FBS medium overnight and treated with activin A (R&D system, 338-AC-010; 25 or 50 ng ml^−1^) for the indicated times.

### Western blotting

The cells were lysed with lysis buffer (50 mM HEPES pH 7.5, 120 mM NaCl, 5 mM EDTA, 10 mM sodium pyrophosphate, 50 mM NaF, 1 mM Na_3_VO_4_ and 1% Triton X-100). The concentration of protein was measured with Bradford protein assay solution (Bio-Red, 5000006) and the same amount of total protein was loaded in SDS–PAGE for western blotting and probed with specific antibodies: anti-PGC1α (NOVUS, NBP1–04676; 1:1,000 dilution), anti-actin (Santa Cruz, sc-517582 horseradish peroxidase; 1:1,000 dilution) or anti-VE-cadherin (Cayman, 160840; 1:1,000 dilution). Quantitative analysis of western blotting was performed using Image J (1.52d, Java 1.8.0_172 (64-bit)).

### Real-time qPCR

Total RNA was isolated by using TriZol Reagent (Invitrogen, 15596026). RT–qPCR was performed as described previously^82^. Briefly, 2 μg of total RNA was used for RT using a high-capacity complementary DNA (cDNA) RT kit (Applied Biosystems, 4368814). qPCR was performed with a fast start universal SYBR green master (ROX) PCR kit (Roche, 04913914001) using QuantStudio7 (Thermo Fisher). Expression of genes was normalized and expressed as the FC relative to *18s* for human genes or to *PPIA* for mouse genes. The sequences of all primers are available in Supplementary Table 8.

### PGC1α promoter luciferase assay

HLMVECs were transfected with 1 μg of a PGC1α promoter luciferase (Addgene, plasmid 8888) and 35 ng of pRL/TK using PEI transfection reagent. Then, 48 h after transfection, the cells were stimulated with vehicle (0.1% BSA), TNF (10 ng ml^−1^), activin A (25 ng ml^−1^) or a combination of TNF and activin A for 16 h and then 100 μl of cell lysate from each sample was used to measure reporter gene expression as described previously^82^. Firefly and *Renilla* luciferase activity was determined by the dual luciferase reagent assay system (Promega). The relative luciferase activity represents the mean value of the firefly/*Renilla* luciferase.

### ChIP assay

Confluent HLMVECs in 100 mm dishes were fixed with 1% PFA for 10 min at room temperature and washed with 1× cold PBS. To quench unreacted PFA, the cells were incubated with 1× glycine for 5 min at room temperature and washed with 1× cold PBS. The nuclear fractions were obtained by using EZ-Magna ChIP A/G kit (Millipore, 17–10086) and then followed by DNA shearing using Covaris (bath temperature, 7.5 °C; acoustic power, 3 W; duty cycle, 2%; time, 60 s; cycle number, 200). The sonicated nuclear fractions were centrifuged at 10,000*g* at 4 °C for 10 min and then a ChIP assay was performed according to manufacturer instructions for the EZ-Magna ChIP A/G kit (Millipore, 17–10086) with minor modifications. Each 50-μl aliquot was used for IP with 1 μg of antibody for normal IgG (negative control), RNA polymerase (positive control) or PGC1α antibody (NOVUS, NBP1–04676) for 3 h. For input control, 5 μl supernatant was aliquoted and stored at 4 °C until protein and DNA elution. The complex of protein and DNA (both input and IP samples) was incubated in elution buffer with proteinase K and RNase A at 37 °C for 30 min, further incubated at 62 °C overnight and then denatured at 95 °C for 10 min. The purified DNA (2 μl) was enriched by qPCR (initial denaturation at 94 °C, 10 min, 1 cycle; denaturation at 94 °C, 20 s; annealing and extension at 60 °C, 1 min, 50 cycles) with primers for putative binding motifs of PPARγ on the *Cdh5* promoter. The gene-amplified values were normalized with input values and presented with the fold enrichment of normal IgG (negative control).

### Bulk RNA-seq and computational data analysis

ECs from skeletal muscles (GC, TA or quadriceps) from mouse hindlimbs were isolated and stained with specific antibodies to CD31 and CD45 and pure muscle ECs (CD31^+^CD45^−^DAPI^−^) were sorted using MoFlo Astrios (Beckman Coulter). The sorted ECs were used for total RNA extraction with TRIzol Reagent (Invitrogen, 15596026). The RNA was treated with DNase and its quality was evaluated with gel quality control. Bulk RNA-seq for 4–6 samples (each sample indicates a mouse) with four time points was performed with oligo-dT mRNA directional using Illumina Novaseq (HiSeq SR50) for coding genes at the Genomic Facility at the University of Chicago. Sequenced reads were aligned to the *Mus musculus* reference genome GRCm39 (mm39) with STAR (version 2.7.6a)^83^. Then, mRNA expression counts were quantified from the aligned reads using the STAR ‘--quantMode’ option. Genes were annotated using the biomaRt R package^84^. We applied the ‘calcNormFactors’ function from the edgeR R package^85^ to normalize the counts. Principal component analysis (PCA) was performed after normalization.

### TrendCatcher temporal gene expression analysis

DDEGs were identified using TrendCatcher^28^ with a threshold of adjusted dynamic *P* value < 0.05. For each gene, its baseline expression fluctuation confidence interval was estimated using negative binomial (NB) distribution. Its nonbaseline gene expression was modeled using a smoothing spline analysis of variance (ANOVA) model with an NB family constraint. For each nonbaseline time point of a gene, the estimated gene expression was tested versus its own baseline fluctuation confidence interval and a dynamic *P* value for each time point was assigned. The gene-wise overall dynamic *P* value was calculated using Fisher’s combined probability test method using all its time dynamic *P* values. Details and benchmarking of the model were described previously^28^. For each DDEG, we calculated its accumulated log_2_ fold change (log_2_FC) over the time compared to its baseline expression. Then, we performed GO enrichment analysis on both positively and negatively accumulated log_2_FC DDEGs using the clusterProfiler R package^86^ and picked the top 10% of most enriched dynamic biological pathways. After removing redundant GO terms for each biological pathway, we calculated the averaged accumulated log_2_FC from its corresponding DDEGs to infer the biological process accumulative change. On the basis of the pathway accumulative change ranking, we selected the top ten positively and negatively changed biological pathways. To show how these GO enrichments change over time, we applied the ‘draw_TimeHeatmap_GO()’ function from TrendCatcher. To build the TimeHeatmap, TrendCatcher first checks all the DDEGs changing direction from their inferred trajectory within each time interval (either up or down). Then, the GO enrichment analysis for both upregulated and downregulated genes was calculated within that time interval. For each GO term within a corresponding time window, a series of log_2_FC gene expression values over time were calculated to quantify the trajectory dynamics of each biological pathway. Then, we subset the TimeHeatmap object using the ‘draw_TimeHeatmap_selGO()’ function to show only the top selective GO terms with the highest accumulative change.

### Single-cell cDNA library preparation

EC^tdTomato^ mice with or without tumors for 3 weeks were used to dissect skeletal muscles (GC, TA or quadriceps) from hindlimbs. The muscle tissues were minced and enzymatically digested with digestion buffer (2 mg ml^−1^ collagenase A, 1 mg ml^−1^ dispase II and 0.1 mg ml^−1^ DNase I in 1× PBS) at 37 °C for 30 min with gentle shaking as mentioned above. Control samples were pooled from three mice and melanoma samples were pooled from four mice. The single-muscle-cell suspension was sorted by tdTomato^high+^DAPI^−^ (muscle ECs and enriched ECs) and tdTomato^−^DAPI^−^ (total mixed muscle tissue cells) using MoFlo Astrios (Beckman Coulter). The sorted cells were loaded into a 10x Genomics microfluidics chip and encapsulated with barcoded oligo-dT-containing gel beads using the 10x Genomics Chromium controller according to the manufacturer’s instructions. Cell viability was more than 90% with a target of sequencing 6,000 cells. Single-cell libraries were constructed using a Chromium single-cell 3′ reagent kit version 3.1 according to the manufacturer’s instructions. The quality of cDNA libraries was checked before sequencing and the cDNA libraries were multiplexed into one lane for sequencing on NovaSeq S1 (28 × 91-bp paired-end reads).

### scRNA-seq data processing and analysis

Raw count tables were generated for each sample with Cell Ranger (version 6.0.2) using default parameters and the 10x mm10–2020-A reference. A total of 24,107 cells passed Cell Ranger quality control (muscle ECs: control tdTomato^+^, 4,046 cells; B16F10 tdTomato^+^, 7,123 cells, muscle tissue cells: control tdTomato^−^, 5,280 cells; B16F10 tdTomato^−^, 7,658 cells). Data analysis was performed using the Scanpy (version 1.9) python package^87^. Cells were filtered and excluded if they had mitochondrial read counts above 20% or if they expressed a gene-by-count ratio within the top 2% or bottom 2% of all cells. Filtered (excluded) cells were annotated as ‘NaN’ in our deposited data. Data were normalized to 10,000 reads per cell using normalize_total() and the highly variable genes were found with highly_variable_genes(). The effects of total counts per cell and mitochondrial counts were regressed out with regress_out(). Each gene was scaled to unit variance and clipped at a value of 10 with the scale() function. PCA was performed using the pca() function on the variable genes. The neighborhood graph was computed using neighbors() with the top 20 principal components. Clustering was performed with the Leiden algorithm then projected to two dimensions using umap(). Corresponding samples were integrated with the ingest() function. For tdTomato^−^ samples, cluster marker genes were found with rank_gene_groups() and cell types were annotated using well-established markers in combination with markers from PangloaDB^33^. Differential expression testing between B16F10 and control cells was performed with the DiffEXpy Python package^88^ using the test.wald() function on the raw normalized counts. Multiple-testing correction was performed using the Benjamini–Hochberg method. DEGs and marker genes were tested for enrichment using the goatools^89^ Python package and all *M.* *musculus* coding genes as the background. Statistically significant enrichments were defined by a corrected *P* value (Benjamini–Hochberg method) ≤ 0.05.

Single-cell hypoxia module scores were calculated using scanpy. tl.score_genes() using the genes from the ‘cellular response to hypoxia’ GO gene set (GO:0071456). The muscle cell differentiation score was likewise calculated using the muscle cell differentiation (GO:0042692) DEGs in bulk EC RNA-seq analysis. The MEME Suite simple enrichment tool^90^ was used to search for JASPAR^91^ vertebrate TF-binding motif enrichment in the promoter regions 1,000 bp upstream and 500 bp downstream of the transcription start sites.

#### Analysis of activin A signaling activity.

We evaluated the activin A signaling activity within EC subpopulations using a set of target genes that are known to be differentially expressed in activin-treated cells (GSE134789)^44^. Each cell was scored with this gene set using scanpy. tl.score_genes() with default parameters.

### Statistics and reproducibility

No statistical methods were used to predetermine sample sizes but our sample sizes are similar to those reported in previous publications^34,41,49^. No data were excluded from the analyses. The experiments were not randomized. The researchers performing experiments with KPC mouse model and the preventive effects of EC PGC1α overexpression in B16F10 and CT26 mice were blinded to the experimental hypothesis. The 3D tissue imaging, IF, western blotting and RT–qPCR assays were performed by researchers who were blinded to the experimental hypothesis. Animals showing signs of unusual illness or severe distress such as huddling, reluctance to move when touched and cold ears were killed and excluded from the analyses.

Quantitative analysis of images and western blotting was performed using ImageJ (National Institutes of Health (NIH); 1.52d, Java 1.8.0_172 (64-bit)) software and Excel (Microsoft). Quantification is presented as the mean ± s.e.m. from at least three independent biological replicate experiments or mice. A Student’s *t*-test with unpaired tests was used for two-group comparisons to determine statistical significance using Prism 9.3.0 GraphPad Software. We also applied a one-way ANOVA for statistical analysis using the aov() function in R followed by post hoc multiple-comparison analysis using a Tukey test. The *P* values were reported and those less than 0.05 were considered statistically significant. Significance levels are indicated in the figures as follows: *P < 0.05, **P < 0.01, ***P < 0.001 and ****P < 0.0001. Data distribution was assumed to be normal but this was not formally tested.

### Reporting summary

Further information on research design is available in the Nature Portfolio Reporting Summary linked to this article.

## Extended Data

## Supplementary Material

Supplementary tables

The online version contains supplementary material available at https://doi.org/10.1038/s43018-025-00975-6.

## Data Availability

All data supporting the findings of this study are available within the paper and Supplementary Information. Bulk RNA-seq data are available from the Gene Expression Omnibus (GEO) (GSE211266). scRNA-seq data are also available from the GEO (GSE211300). Source data are provided with this paper.
