# Supplementary material for: Skeletal muscle endothelial dysfunction through the activin A–PGC1α axis drives progression of cancer cachexia
Source: Nat Cancer. 2025 May 26;6(8):1350–69. doi: 10.1038/s43018-025-00975-6 (PMC12254930; doi:10.1038/s43018-025-00975-6)
Supplement: Supplementary file 1 — Reporting Summary [file 43018_2025_975_MOESM1_ESM.pdf]

nature portfolio

Corresponding author(s): Young-Mee Kim,Jalees Rehman

Last updated by author(s): Mar 21, 2025

Reporting Summary

Nature Portfolio wishes to improve the reproducibility of the work that we publish. This form provides structure for consistency and transparency in reporting. For further information on Nature Portfolio policies, see our Editorial Policies and the Editorial Policy Checklist.

Statistics

For all statistical analyses, confirm that the following items are present in the figure legend, table legend, main text, or Methods section.

n/a	Confirmed	
☐	☒	The exact sample size (*n*) for each experimental group/condition, given as a discrete number and unit of measurement
☐	☒	A statement on whether measurements were taken from distinct samples or whether the same sample was measured repeatedly
☐	☒	The statistical test(s) used AND whether they are one- or two-sided<br>*Only common tests should be described solely by name; describe more complex techniques in the Methods section.*
☒	☐	A description of all covariates tested
☐	☒	A description of any assumptions or corrections, such as tests of normality and adjustment for multiple comparisons
☐	☒	A full description of the statistical parameters including central tendency (e.g. means) or other basic estimates (e.g. regression coefficient) AND variation (e.g. standard deviation) or associated estimates of uncertainty (e.g. confidence intervals)
☐	☒	For null hypothesis testing, the test statistic (e.g. *F*, *t*, *r*) with confidence intervals, effect sizes, degrees of freedom and *P* value noted<br>*Give P values as exact values whenever suitable.*
☒	☐	For Bayesian analysis, information on the choice of priors and Markov chain Monte Carlo settings
☒	☐	For hierarchical and complex designs, identification of the appropriate level for tests and full reporting of outcomes
☒	☐	Estimates of effect sizes (e.g. Cohen's *d*, Pearson's *r*), indicating how they were calculated

Our web collection on statistics for biologists contains articles on many of the points above.

Software and code

Policy information about availability of computer code

Data collection	Microscopy images were acquired using ZEN blue or black by LSM 710, LSM900, or Aperio imageScope v12.4.6. Flow cytometry data was collected using Kaluza software 2.2.1. qPCR data was collected using QuantStudio7 (Thermo Fisher) or CFX Duet real time PCR system (BioRad). Western blot images were collected using iBright CL1500 Imaging system (ThermoFisher) or Azure Western Blot Imaging Systems.
Data analysis	The confocal images were analyzed with Image J (ImageJ bundled with 64-bit Java 1.8.0_172, NIH), maris X64 version 9.5.0 (Bitplane Scientific) and Zeiss ZEN blue 3.9 software. The Western blots were analyzed with Image J (NIH). FACS data was analyzed with Kaluza software 2.2.1 (Beckman Coulter). Raw data from technical replicates were analysed using Excel (Microsoft) prior to statistical analysis of biological replicates using GraphPad Prism9.<br><br>The genes from bulk RNAseq were annotated using biomaRt R package and edgeR R package to normalize counts.<br>The dynamic differentially expressed genes were identified using TrendCacher. The TrendCatcher software platform used for longitudinal analysis of transcriptomic data is available at: https://github.com/jaleesr/TrendCatcher.<br><br>The scRNAseq data analysis was performed using Scanpy(v1.9) python package.<br>The MEME Suite Simple Enrichment tool (https://www.biorxiv.org/content/10.1101/2021.08.23.457422v1) was used to search for JASPAR (https://doi.org/10.1093/nar/gkab1113) vertebrate transcription factor binding motif enrichment .

For manuscripts utilizing custom algorithms or software that are central to the research but not yet described in published literature, software must be made available to editors and reviewers. We strongly encourage code deposition in a community repository (e.g. GitHub). See the Nature Portfolio guidelines for submitting code & software for further information.

Data

Policy information about availability of data

All manuscripts must include a data availability statement. This statement should provide the following information, where applicable:

- Accession codes, unique identifiers, or web links for publicly available datasets
- A description of any restrictions on data availability
- For clinical datasets or third party data, please ensure that the statement adheres to our policy

The authors declare that the all data supporting the findings of this study are available within the paper and in its extended data files.

Bulk RNA-seq data is available at the following NCBI GEO link:
https://www.ncbi.nlm.nih.gov/geo/query/acc.cgi?acc=GSE211266

Single-cell RNA-seq data is available at the following NCBI GEO link:
https://www.ncbi.nlm.nih.gov/geo/query/acc.cgi?acc=GSE211300

The TrendCatcher software platform used for longitudinal analysis of transcriptomic data is available at: https://github.com/jaleesr/TrendCatcher.

We used gene sets:Cellular response to hypoxia' GO gene set (GO:0071456), muscle cell differentiation (GO:0042692) genes, and Activin-treated cells (GSE134789).

Human research participants

Policy information about studies involving human research participants and Sex and Gender in Research.

Reporting on sex and gender	Our collection of human samples was deemed to not constitute human subjects research by the UIC Office for the Protection of Research Subjects because we obtained muscle sections from de-identified FFPE blocks from the UIC Pathology Biorepository.<br>Data was not collected via interactions/interventions with individuals for research purposes, and there was no use of private, identifiable information about subjects. We have provided the gender of patients at Supplementary Table 7.
Population characteristics	See above.
Recruitment	N/A
Ethics oversight	Our collection of human samples was deemed to not constitute human subjects research by the UIC Office for the Protection of Research Subjects because we obtained muscle sections from de-identified FFPE blocks from the UIC Pathology Biorepository. We did not collect data via interactions/interventions with individuals for research purposes, and use any private, identifiable information about subjects.

Note that full information on the approval of the study protocol must also be provided in the manuscript.

Field-specific reporting

Please select the one below that is the best fit for your research. If you are not sure, read the appropriate sections before making your selection.

☒ Life sciences ☐ Behavioural & social sciences ☐ Ecological, evolutionary & environmental sciences

For a reference copy of the document with all sections, see nature.com/documents/nr-reporting-summary-flat.pdf

Life sciences study design

All studies must disclose on these points even when the disclosure is negative.

Sample size	No statistical methods were used to determine sample size. The sample size were determined based on the variability of previously published experiments (Fan, Z. et al. Cell Metab 33, 1793-1807 (2021), Hulmi, J. J. et al. Mol Metab 41, 101046 (2020), Taylor, J. et al. Nat Cancer 4, 1544-1560 (2023)). All sample sizes are indicated in the figure legends.
Data exclusions	Data were excluded if technical problems were detected (severe deviation in technical replicates). Some tumor bearing mice or KPC mice were excluded from experiments if they had to sacrificed early for ethical reasons. All repetitions of experiments were consistent.
Replication	We performed at least three independent biological replicate experiments. For each of these biological replicates, we used additional technical replicates for protein quantification assay and qPCR to ensure the reproducibility of our studies. When representative images were shown, they were based on at least three independent replicate experiments. All attempts at replication were consistent. For scRNAseq analysis, samples from 3-4 mice were pooled.

Randomization

No randomization as these were animal and cell based studies. No statistical methods were used to pre-determine sample sizes but our sample sizes are similar to those reported in previous publications.

Blinding

The tested animals had ear tags. We performed all experiments while being blinded to the animal genotype. After completion of experiments, the genotype was revealed and the data was analyzed according to the genotype. Blinding was used during analysis where the data were quantified/measured blind to the treatments. The researchers performing experiments with KPC mouse model and the preventive effects of EC-PGC1α overexpression in melanoma and CT26 bearing mice were blinded to the experimental hypothesis. The 3D tissue imaging, IF, Western Blotting and RT-qPCR assays were performed by researchers who were blind to the experimental hypothesis. In all other experiments, data collection and analyses were not blinded to the condition of the experiments.

Reporting for specific materials, systems and methods

We require information from authors about some types of materials, experimental systems and methods used in many studies. Here, indicate whether each material, system or method listed is relevant to your study. If you are not sure if a list item applies to your research, read the appropriate section before selecting a response.

Materials & experimental systems

n/a	Involved in the study
☐	☒ Antibodies
☐	☒ Eukaryotic cell lines
☒	☐ Palaeontology and archaeology
☐	☒ Animals and other organisms
☒	☐ Clinical data
☒	☐ Dual use research of concern

Methods

n/a	Involved in the study
☒	☐ ChIP-seq
☐	☒ Flow cytometry
☒	☐ MRI-based neuroimaging

Antibodies

Antibodies used

All antibodies are from commercial sources. Detailed infromation on their use is provided within Method section and step-by-step protocol.
Flow cytometric analysis: CD45 (157610, BioLegend, 1:100), CD31 (eBiosciences 17-0311-82, 1:100),CD45 (BioLegend 103108, 1:100)
Muscle 3D imaging: CD31 (BioLegend, 102502, 1:1000),anti-rat IgG2 antibody (BioLegend, 407502, 1:100), DyLight 633 (Thermofisher, 46414, 1:10)

In vivo tissue immunofluorescence assay: CD31 antibody (BD biosciences, 550274, 1:25), laminin (Sigma, L9393, 1:100), goat anti-Rat IgM DyLight 488 (Thermo Fisher, SA5-10010, 1:400), goat anti-Rabbit IgG (H+L) Alexa fluor 633 (Thermo Fisher, A21070, 1:500), CD31 antibody (abcam, ab28364, 1:100), biotinylated isolectin B4 (Vector lab, B-1205-.5, 1:100), goat anti-rabbit Alexa Fluor594 (A-11012, Invitrogen, 1:500) or FITC-streptavidin (Invitrogen, 11-4317-87, 1:500), type 1 for slow oxidative fiber (DSHB, BA-D5, 1:50), type 2a for fast oxidative fiber (DSHB, sc-71, 1:100), type 2b for fast glycolytic fiber (DSHB, BF-F3, 1:50), goat anti-mouse IgG2b Alexa Fluor546 secondary antibody (Invitrogen, A-21143, 1:400), rat anti-mouse IgG1-FITC secondary antibody (Invitrogen, 11-4015-82, 1:400), goat anti-mouse IgM (heavy chain) Alexa Fluor633 secondary antibody (A21046, 1:400), or goat anti-mouse IgM DyLight650 secondary antibody (SA5-10153, 1:400), mouse anti-human CD31 antibody (Endothelial Cell Clone JC70A, M0823, Dako, 1:100), rabbit anti-VE-cadherin (Cayman, #160840, 1:100), goat anti-mouse Alexa Fluor488 secondary antibody (A-21121, Invitrogen, 1:500), goat anti-rabbit Alexa Fluor647 secondary antibody (A-21245, Invitrogen, 1:500), ERG1 (abcam, AB92513, 1:100), SMA (abcam, AB5694, 1:100)

In vitro cell immunofluorescene assay: Alexa Fluor647 mouse anti-human CD144 (BD561567, 1:250)

Western Blotting: PGC1α (NOVUS, NBP1-04676, 1:1000), Actin (Santa Cruz, sc-517582 HRP, 1:1000), or VE-cadherin (Cayman #160840, 1:1000), pFOXO-1/3/4 (Cell Signaling, 2599T, 1:1000), FOXO-1 (Cell Signaling, 2880T, 1:1000), FOXO-3 (Cell Signaling, 12829, 1:1000), FOXO-4 (Cell Signaling, 9472T, 1:1000), P-SMAD2/3(Cell Signaling, 8828, 1:1000), or P-SMAD3 (NOVUS, NBP1-77836SS, 1:1000).

In vivo experiments: anti-Activin-A neutralizing antibody (R&D systems, MAB3381), normal IgG (R&D systems, MAB002)

Validation

Antibodies were validated by the commercial vendors and published papers. We additional validated the antibodies ourselves using Western blotting, immunoprecipitation, or immunofluorescence imaging. The detail number of citation according to CiteAb website is follow: CD31 (BioLegend 102502, eBiosciences 17-0311-82, and BD biosciences 550274) was broadly validated (75, 145 and 1345 citations). CD45 (BioLegend 103108 and 157610) was broadly validated (228 and 9 citations). anti-rat IgG2 (BioLegend, 407502) was broadly validated (2 citations). Laminin (Sigma, L9393) was broadly validated (1674 citations). goat anti-Rabbit IgG (H+L) cross-adsorbed secondary antibody Alexa fluor 633 (Thermo fisher, A21070) was broadly validated (479 citations). CD144 (BD561567) was broadly validated (19 citations). PGC1α (NOVUS, NBP1-04676) was broadly validated (152 citations). Actin (Santa Cruz, sc-517582 HRP) was broadly validated (78 citations). VE-cadherin (Cayman #160840) was broadly validated (26 citations).FOXO-1 (Cell Signaling, 2880T) was broadly validated (8 citations). FOXO-3 (Cell Signaling, 12829) was broadly validated (204 citations).FOXO-4 (Cell Signaling, 9472T) was validated (1citation). P-SMAD2/3(Cell Signaling, 8828) was broadly validated (675 citations). P-SMAD3 (NOVUS, NBP1-77836SS) was validated (3 citations). ERG1 (AB92513) was broadly validated (282 citations). SMA (AB5694)was broadly validated (3352 citations). Myosin heavy chain Type IIA (sc-71, DSHB) was broadly validated (847 citations). Myosin heavy chain Type

IIB (BF-F3, DSHB) was broadly validated (627 citations). Myosin heavy chain Type I (BA-D5, DSHB) was broadly validated (403 citations).

Eukaryotic cell lines

Policy information about cell lines and Sex and Gender in Research

Cell line source(s)	We purchased human lung micro-vascular endothelial cells (#CC-2527) from Lonza. We purchased HEK293T (CRL-11268), B16F10 (CRL-6475), CT26 (CRL-2639) and LLC1 (CRL-1642) from ATCC. We did not consider the cell lines biological sex in the study design.
Authentication	We used primary endothelial cells that are isolated by the vendor and authenticated by the vendor.<br>We additionally performed Western blotting to validate their endothelial phenotype.
Mycoplasma contamination	The vendors test negative for mycoplasma, bacteria, yeast, and fungi.
Commonly misidentified lines<br>(See ICLAC register)	We did not use any commonly misidentified cell lines for our experimental studies.

Animals and other research organisms

Policy information about studies involving animals; ARRIVE guidelines recommended for reporting animal research, and Sex and Gender in Research

Laboratory animals	C57/BL6, BALB/cJ, tdTomato flfl:Cdh5-CreERT2, PGC1α flfl, PGC1α flfl:tdTomato flfl:Cdh5-CreERT2, 8-10weeks age, female and male. KPC mice 3-5 month age, female and male. We used both male and female mice but did not consider sex dependency for this project. Housing condition: Mice were maintained under standard conditions (standard diet and water) at 23 °C and ~60% humidity with 12 h light and 12 h dark cycles.
Wild animals	The study did not involve wild animals.
Reporting on sex	We used male and female mice in this study. We did not performed between sex comparative analyses.
Field-collected samples	The study did not involve samples collected from the field.
Ethics oversight	All animal studies were carried out following protocols approved by the Animal Care and Institutional Biosafety Committee of the University of Illinois Chicago (ACC #24-084). The Biological Resources Laboratory is fully accredited by the American Association for Accreditation of Laboratory Animal Care (AAALAC). All aspects of working with mice, including procurement, quarantine, housing, management, veterinary care and disposal of carcasses follow the guidelines set down in the NIH Guide for the Care and Use of Laboratory Animals.

Note that full information on the approval of the study protocol must also be provided in the manuscript.

Flow Cytometry

Plots

Confirm that:

☒ The axis labels state the marker and fluorochrome used (e.g. CD4-FITC).

☒ The axis scales are clearly visible. Include numbers along axes only for bottom left plot of group (a 'group' is an analysis of identical markers).

☒ All plots are contour plots with outliers or pseudocolor plots.

☒ A numerical value for number of cells or percentage (with statistics) is provided.

Methodology

Sample preparation	Single cell suspensions were isolated from skeletal muscle tissue of KPC and tumor bearing C57BL6 mice using enzymatic digestion.
Instrument	Gallios flow cytometer (Beckman Coulter, Pasadena, CA)
Software	Kaluza software (Beckman Coulter)
Cell population abundance	We determined the relevant cell population based on cell surface markers such as CD31 positive or CD45 positive. Each cell population is presented as % of whole tissue.

Gating strategy

Single cells isolated from skeletal muscle tissue were gated on CD31 positive (for endothelial cells) or CD45 positivity (for immune cells) with DAPI negative.

☒ Tick this box to confirm that a figure exemplifying the gating strategy is provided in the Supplementary Information.
