# Supplementary figures and images for: Skeletal muscle endothelial dysfunction through the activin A–PGC1α axis drives progression of cancer cachexia
Source: Nat Cancer. 2025 May 26;6(8):1350–69. doi: 10.1038/s43018-025-00975-6 (PMC12254930; doi:10.1038/s43018-025-00975-6)

**Fig. 6a**

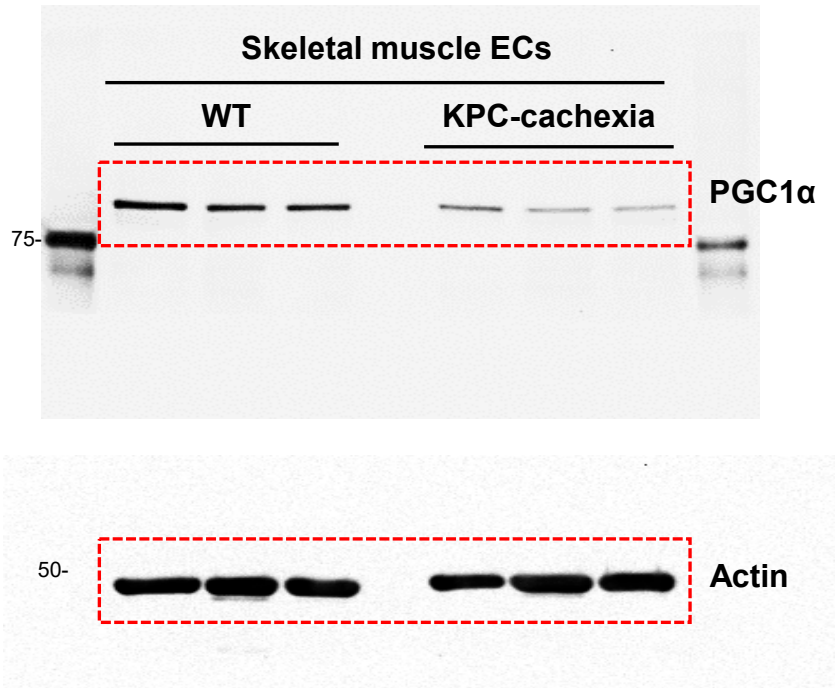

**Fig. 7a**

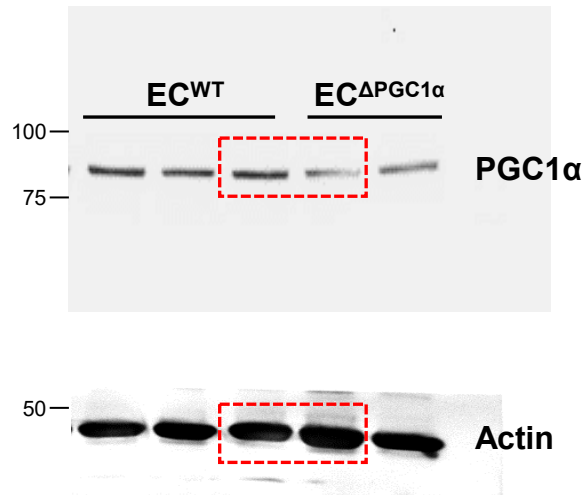

ED Fig. 3f

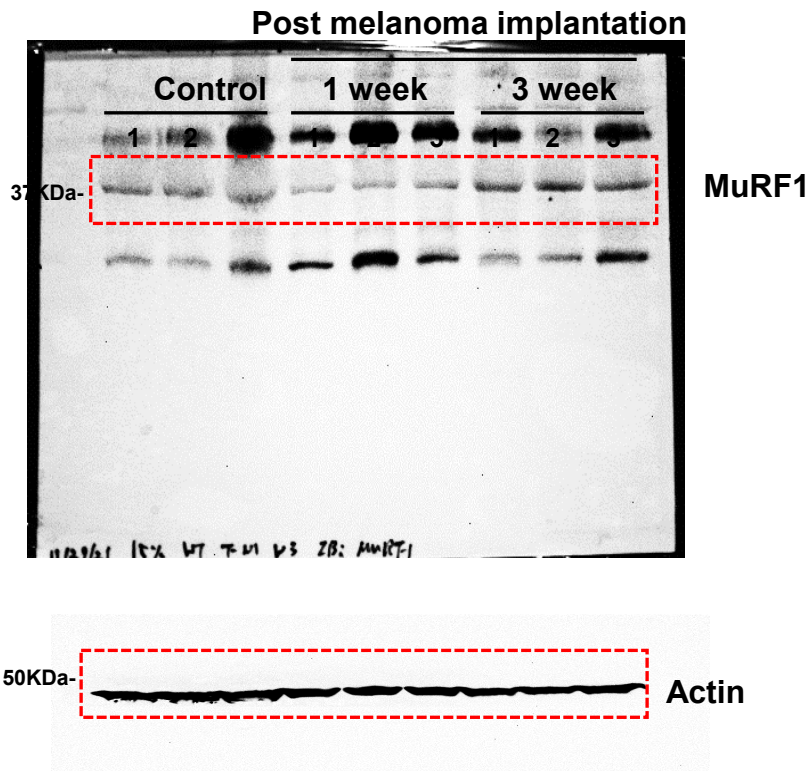

**ED Fig. 6i**

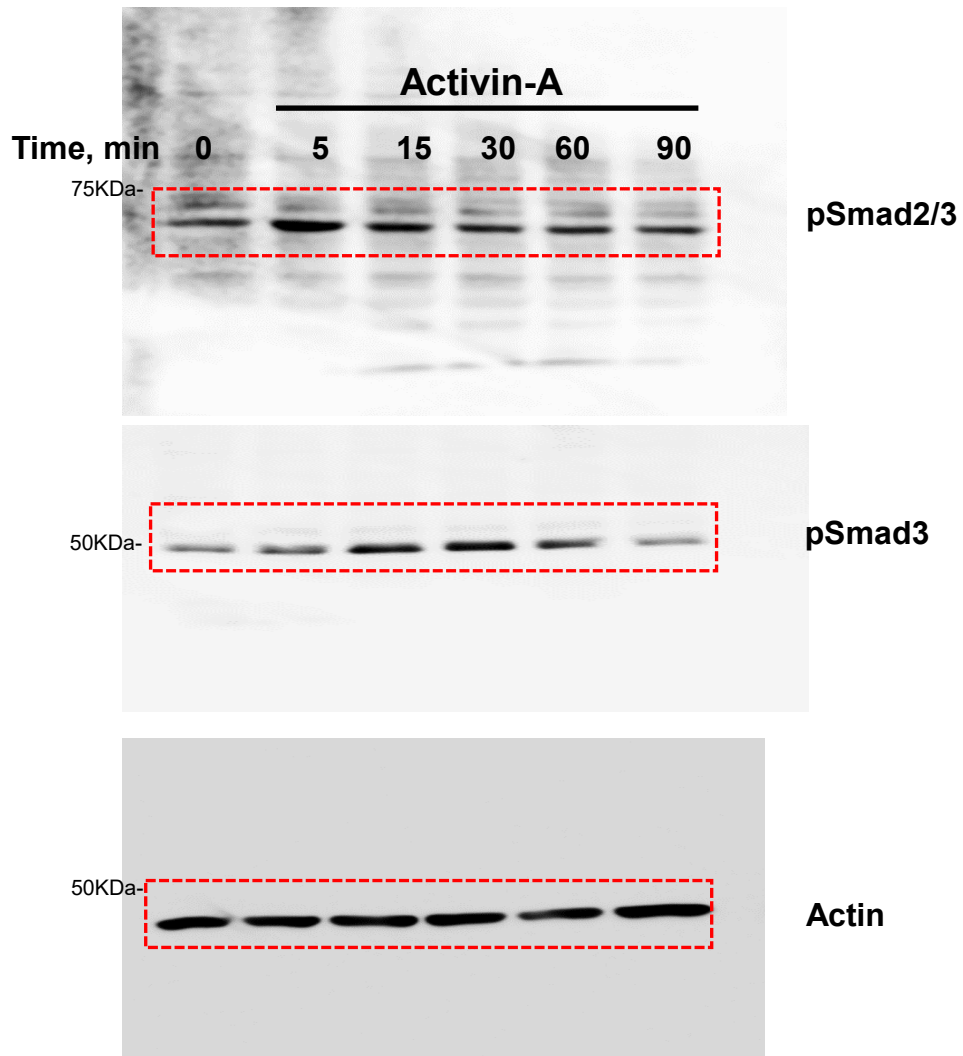

**ED Fig. 7k**

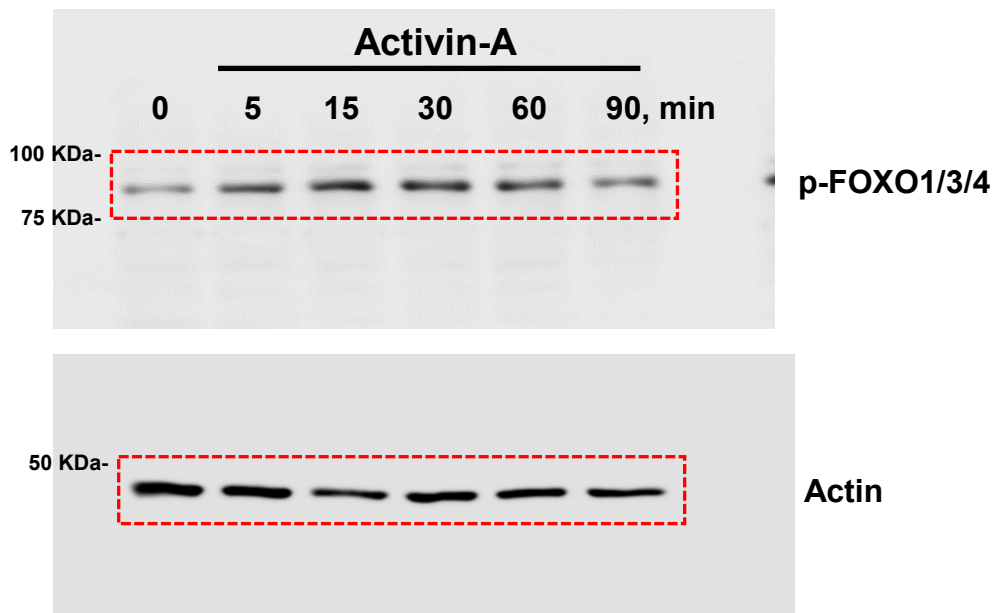

## ED Fig. 7m

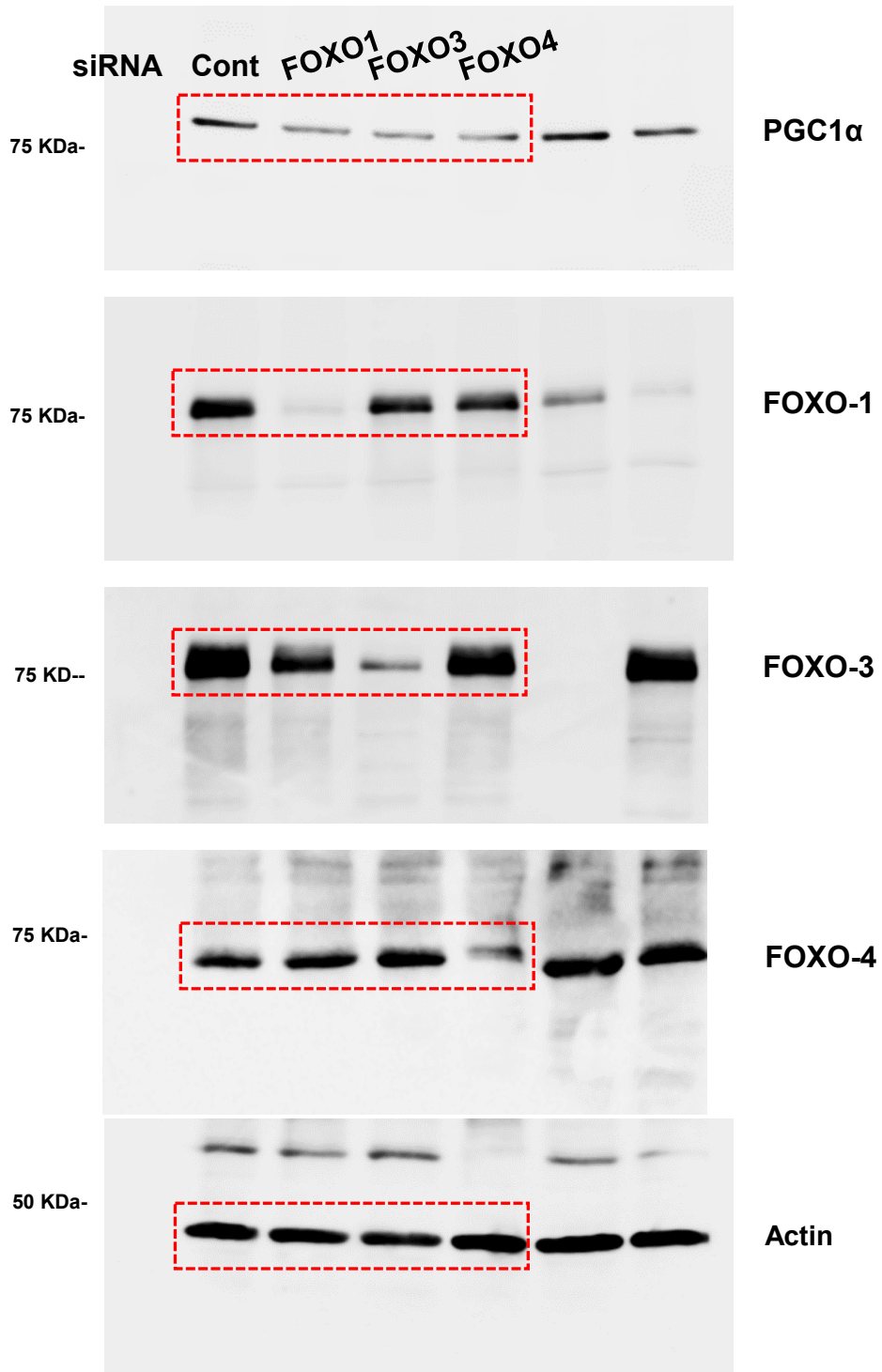

## ED Fig. 8d

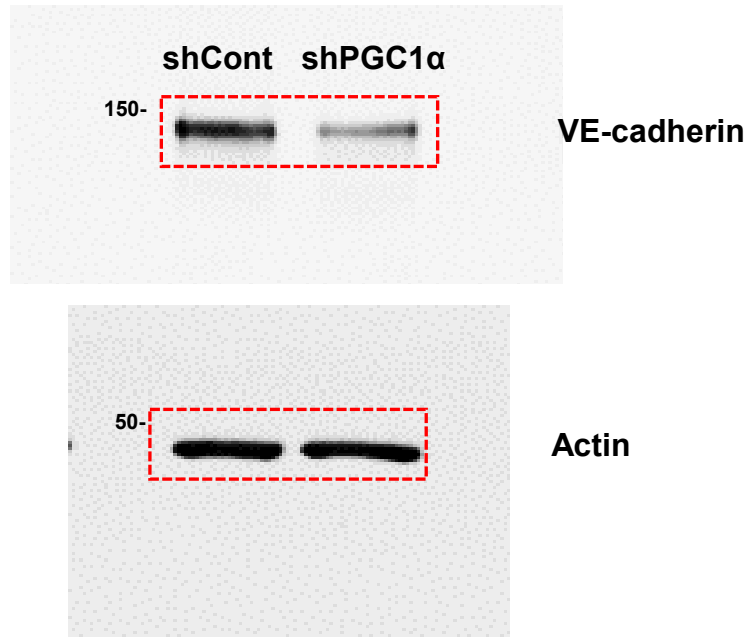

## ED Fig. 8i

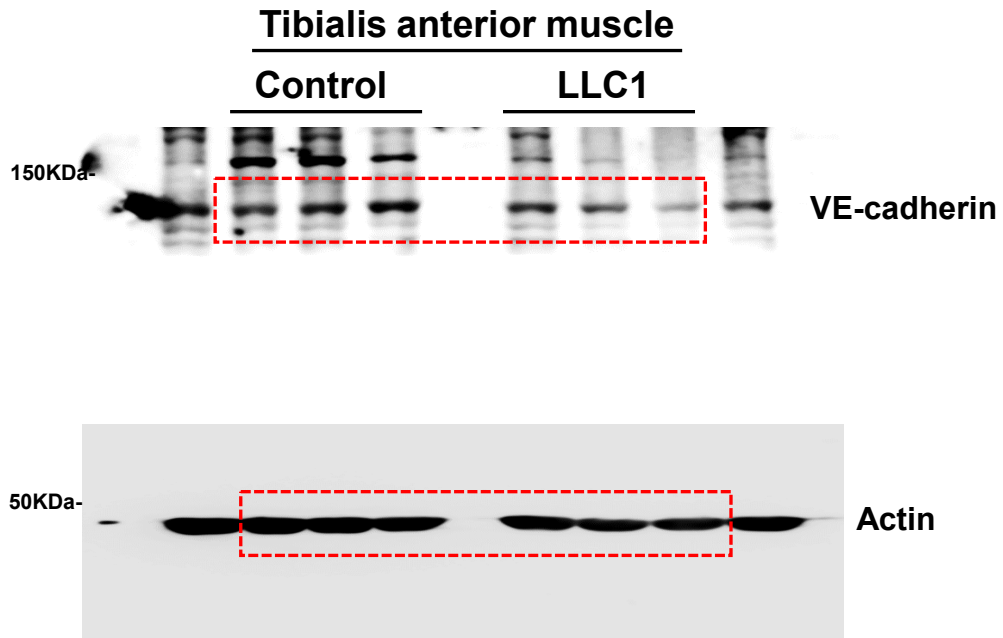

Supplement: Supplementary file 21 — Unprocessed gels or blots. [file 43018_2025_975_MOESM21_ESM.pdf]
